# Kinase SnRK1.1 regulates nitrate channel SLAH3 engaged in nitrate-dependent alleviation of ammonium toxicity

**DOI:** 10.1093/plphys/kiab057

**Published:** 2021-02-09

**Authors:** Doudou Sun, Xianming Fang, Chengbin Xiao, Zhen Ma, Xuemei Huang, Jingrong Su, Jia Li, Jiafeng Wang, Suomin Wang, Sheng Luan, Kai He

**Affiliations:** 1 Ministry of Education Key Laboratory of Cell Activities and Stress Adaptations, School of Life Sciences, Lanzhou University, Lanzhou 730000, China; 2 State Key Laboratory of Grassland Agro-ecosystems, Lanzhou University, Lanzhou 730020, China; 3 Key Laboratory of Grassland Livestock Industry Innovation, Ministry of Agriculture and Rural Affairs, Lanzhou University, Lanzhou 730020, China; 4 College of Pastoral Agriculture Science and Technology, Lanzhou University, Lanzhou 730020, China; 5 Department of Plant and Microbial Biology, University of California, Berkeley, California 94720, USA

## Abstract

Nitrate (${\text{NO}}_{3}^{-}$) and ammonium (${\text{NH}}_{4}^{+}$) are major inorganic nitrogen (N) supplies for plants, but ${\text{NH}}_{4}^{+}$ as the sole or dominant N source causes growth inhibition in many plants, known as ammonium toxicity. Small amounts of ${\text{NO}}_{3}^{-}$ can significantly mitigate ammonium toxicity, and the anion channel SLAC1 homolog 3 (SLAH3) is involved in this process, but the mechanistic detail of how SLAH3 regulates nitrate-dependent alleviation of ammonium toxicity is still largely unknown. In this study, we identified SnRK1.1, a central regulator involved in energy homeostasis, and various stress responses, as a SLAH3 interactor in Arabidopsis (*Arabidopsis thaliana*). Our results suggest that SNF1-related protein kinase 1 (SnRK1.1) functions as a negative regulator of SLAH3. Kinase assays indicate SnRK1.1 strongly phosphorylates the C-terminal of SLAH3 at the site S601. Under high-${\text{NH}}_{4}^{+}$/low-pH condition, phospho-mimetic and phospho-dead mutations in *SLAH3 S601* result in barely rescued phenotypes and fully complemented phenotypes in *slah3*. Furthermore, SnRK1.1 migrates from cytoplasm to nucleus under high-${\text{NH}}_{4}^{+}$/low-pH conditions. The translocation of SnRK1.1 from cytosol to nucleus under high-ammonium stress releases the inhibition on SLAH3, which allows SLAH3-mediated ${\text{NO}}_{3}^{-}$ efflux leading to alleviation of high-${\text{NH}}_{4}^{+}$/low-pH stress. Our study reveals that the C-terminal phosphorylation also plays important role in SLAH3 regulation and provides additional insights into nitrate-dependent alleviation of ammonium toxicity in plants.

## Teaser

Nitrate-dependent alleviation of ammonium toxicity involves negative regulation of a nitrate channel by a kinase.

## Introduction

Nitrogen (N) is an essential nutrient element for all living organisms on earth. For plants, N is an indispensable component of proteins, nucleic acids, and chlorophylls. Except for legumes, most plants are not capable of directly utilizing the gaseous N abundant in the atmosphere and can only take up organic forms of N such as amino acids (AAs) and inorganic forms of N mainly including ammonium (${\text{NH}}_{4}^{+}$) and nitrate (${\text{NO}}_{3}^{-}$) through specific transportation systems in root (Ludewig et al., 2007; Wang et al., 2012).

In Arabidopsis (*Arabidopsis thaliana*), the uptake of ${\text{NH}}_{4}^{+}$ is mediated by ammonium transporters (AMTs) localized at the plasma membrane (PM; Ludewig et al., 2007). When the root absorbs ${\text{NH}}_{4}^{+}$ from the environment, the AMT-coupled H^+^-ATPase pumps protons out of the root cell in order to maintain balanced intracellular charge and stabilize membrane potential (Meier et al., 2020; Motte and Beeckman, 2020). ${\text{NO}}_{3}^{-}$ is transported by PM-localized nitrate transporters (NRTs; Wang et al., 2012). Unlike AMT-mediated ${\text{NH}}_{4}^{+}$ absorption that often causes H^+^ efflux, most NRTs are ${\text{NO}}_{3}^{-}$/H^+^ symporters, mediating ${\text{NO}}_{3}^{-}$ uptake accompanied by H^+^ influx (Mengel et al., 1983; Pearson and Stewart, 1993; Wang et al., 2012; Xu et al., 2012).

After absorption, ${\text{NO}}_{3}^{-}$, the oxidized form of inorganic N, needs to be reduced to ${\text{NH}}_{4}^{+}$ before further assimilation, catalyzed by nitrate reductase and nitrite reductase (Crawford, 1995). Therefore, ${\text{NH}}_{4}^{+}$ is theoretically the preferred inorganic N source for plants (Salsac et al., 1987). However, when ${\text{NH}}_{4}^{+}$ is the dominant N source, it often significantly inhibits plant growth, featured by the phenotypes of severely inhibited root growth and chlorosis, known as ammonium toxicity (Kronzucker et al., 1997; Britto and Kronzucker, 2002). To date, little is known about the detailed molecular mechanisms of ammonium toxicity. Nevertheless, nitrate effectively alleviates ammonium toxicity (Domene and Ayres, 2001; Britto and Kronzucker, 2002; Houdusse et al., 2005). Likewise, how nitrate lessens ammonium toxicity is largely unclear.

The proteins in slow anion channel/SLAC1 homolog (SLAC/SLAH) family in vascular plants function as efflux channels for anions such as Cl^−^ and ${\text{NO}}_{3}^{-}$ (Geiger et al., 2009; Wang et al., 2012). In Arabidopsis, the *SLAC*/*SLAH* gene family consists of five members, *SLAC1* and *SLAH1* to *4*, encoding PM proteins with predicted 10 transmembrane helixes with both amino termini (NT) and carboxyl termini (CT) in cytoplasm (Negi et al., 2008; Vahisalu et al., 2008; Hedrich and Geiger, 2017). *SLAC1* is specifically expressed in guard cells and its encoding protein product is an important regulator of stomatal movement (Negi et al., 2008; Vahisalu et al., 2008). SLAH3 plays the same role as SLAC1 in regulating stomatal movement (Geiger et al., 2011). In guard cells, the channel activity of SLAH3 is regulated by reversible phosphorylation mediated by protein kinase CPK21 and protein phosphatase ABI1 (Geiger et al., 2011; Demir et al., 2013). Compared to SLAC1, SLAH3 has a stronger selectivity for ${\text{NO}}_{3}^{-}$, with an about 20 times higher affinity over Cl^−^, and is therefore considered as a nitrate efflux channel (Geiger et al., 2011; Wang et al., 2012).

*SLAH3* is also expressed in roots, suggesting it may serve distinct physiological functions in addition to regulating stomatal movement (Zheng et al., 2015). Our previous studies show that *slah3* mutants are extremely sensitive to the culture conditions with higher concentrations of ${\text{NH}}_{4}^{+}$ and lower concentrations of ${\text{NO}}_{3}^{-}$ compared with wild-type (WT) Columbia-0 (Col-0) plants (Zheng et al., 2015). We also found that under high-ammonium conditions, elevating the medium pH significantly lessens ammonium toxic phenotypes in plants, and more importantly, wipes out the phenotypic differences between *slah3* mutants and Col-0. These results indicate medium acidification is likely one of the important causes of ammonium toxicity and that SLAH3 may function through regulating the pH of the rhizosphere. Our study thus revealed SLAH3 is involved in the process of nitrate-dependent alleviation of ammonium toxicity likely via mediating nitrate efflux under high-${\text{NH}}_{4}^{+}$/low-pH conditions (Zheng et al., 2015). We speculate that under high-${\text{NH}}_{4}^{+}$ conditions, the absorption of ${\text{NH}}_{4}^{+}$ leads to accumulation of apoplastic H^+^, causing rapid acidification in the rhizosphere. In response to this condition, SLAH3 mediates the outflow of ${\text{NO}}_{3}^{-}$ to the rhizosphere. ${\text{NO}}_{3}^{-}$/H^+^ symporter NRTs then mediate influx of both ${\text{NO}}_{3}^{-}$ and H^+^, reducing the medium acidification caused by absorption of ${\text{NH}}_{4}^{+}$ and effectively alleviating ammonium toxicity.

Sucrose non-fermenting 1 (SNF1)-related protein kinase 1 (SnRK1) proteins, the paralogs of SNF1 in yeasts and AMP-activated protein kinases (AMPK) in animals, are a group of Ser/Thr protein kinases widely found in plants (Polge and Thomas, 2007; Crozet et al., 2014). SnRK1/SNF1/AMPK kinases play central roles in sensing sugar signals and regulating energy homeostasis (Baena-González and Sheen, 2008; Crozet et al., 2014). In plants, SnRK1s also play key roles in regulating biotic and abiotic stress responses as well as plant growth and development (Baena-González et al., 2007; Polge and Thomas, 2007; Baena-González and Sheen, 2008; Hulsmans et al., 2016). SnRK1 is a heterotrimeric complex composed of a catalytic subunit and two regulatory subunits (Crozet et al., 2014; Hulsmans et al., 2016). The catalytic subunit is encoded by *SnRK1.1* (*KIN10*) or *SnRK1.2* (*KIN11*) in Arabidopsis. *SnRK1.1* and *SnRK1.2* are highly functionally redundant. Single mutation in *SnRK1.1* or *SnRK1.2* causes no obvious phenotypic defects but the *snrk1.1 snrk1.2* double mutant is lethal (Baena-González et al., 2007).

In plants, the *SnRK1* family has expanded to a larger family containing *SnRK2* and *SnRK3* subfamilies (Halford and Hardie, 1998; Hrabak et al., 2003). SnRK2s mainly function in ABA signaling pathway and SnRK3s, also known as calcineurin B-like (CBL)-interacting protein kinases (CIPKs), interact with CBL proteins upon Ca^2+^ signal to regulate various stress responses (Fujii and Zhu, 2009; Luan, 2009; Coello et al., 2011). In plants, calcium-dependent protein kinases (CPKs) share similar structures with the SnRK family and are included in the CPK–SnRK superfamily (Hrabak et al., 2003). Intriguingly, a number of CPK–SnRK members have been identified as key regulators in controlling the activities of ion transporters and channels (Kudla et al., 2018).

To understand the molecular mechanisms of how SLAH3 is regulated in response to high-${\text{NH}}_{4}^{+}$ stress, we identified SnRK1.1 as a SLAH3 interactor through a yeast-two-hybrid screen. Distinct from the previously identified kinases that associate with and phosphorylate the NT of SLAH3 and subsequently activate the SLAH3 channel, SnRK1.1 interacts with and phosphorylates the CT of SLAH3. By using a combination of molecular, biochemical, and genetic approaches, we demonstrated that SnRK1.1 likely negatively regulates SLAH3 in modulating the process of nitrate-dependent alleviation of ammonium toxicity. Moreover, we found that SnRK1.1 translocates from cytoplasm to nucleus upon high-ammonium conditions, suggesting the release of the inhibition of SnRK1.1 on SLAH3 in response to high-ammonium conditions. Thus, our study revealed an additional mechanism of how nitrate efflux channel SLAH3 is regulated in response to high-ammonium stress.

## Results

### Protein kinase SnRK1.1 interacts with the C-terminus of SLAH3

**Figure 1:**
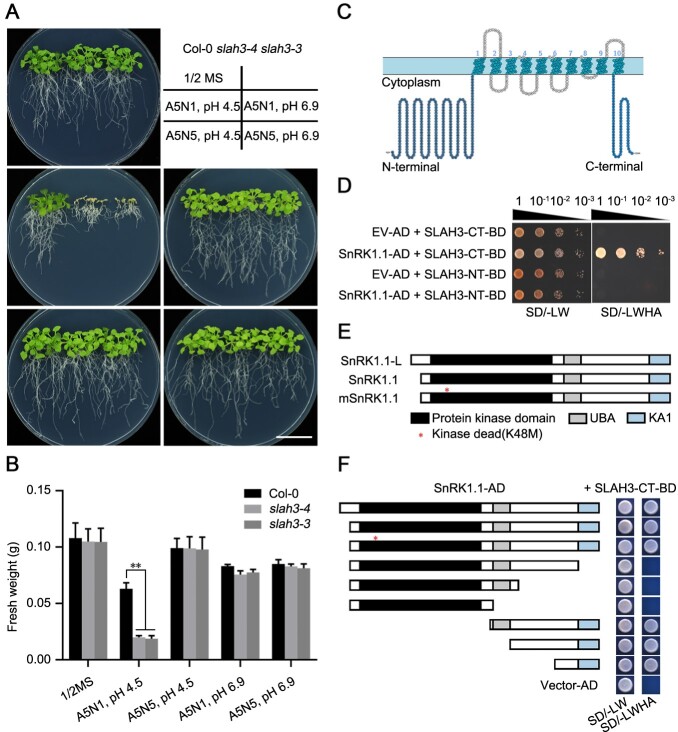
SnRK1.1 interacts with the C-terminal of SLAH3. A, *slah3* mutants are hyper-sensitive to high-ammonium conditions. Col-0, *slah3-3* and *slah3-4* plants grown for 20 d under different culture conditions are presented. 5 mM ${\text{NH}}_{4}^{+}$, 1 mM ${\text{NO}}_{3}^{-}$ (A5N1); 5 mM ${\text{NH}}_{4}^{+}$, 5 mM ${\text{NO}}_{3}^{-}$ (A5N5). White scale = 2 cm. B, The statistical analyses of fresh weight on the plants presented in (A). Data are shown as means ± se, *n* = 3. Statistical analyses were performed by two-way ANOVA analysis with Dunnett’s multiple comparisons test. *P < 0.05; **P < 0.01. C, Predicted topology of the SLAH3 monomer. Cartoon of SLAH3 protein predicted by http://wlab.ethz.ch/protter/start/ is shown. Each circle represents an AA. SLAH3 contains 10 transmembrane helixes with both N-terminal and C-terminal in the cytoplasm. D, SnRK1.1 interacts with the C-terminal of SLAH3 but not the N-terminal of SLAH3. The growth of the yeast cells co-transformed with various combinations of the plasmids were analyzed on synthetic dropout medium lacking Leu and Trp (-L-W) and synthetic dropout medium lacking Leu, Trp, His, and adenine (-L-W-H-A). E, SnRK1.1 protein contains a serine/threonine kinase domain, an UBA domain, and a C-terminal kinase-related (KA1) domain. SnRK1.1-Long (L) (At3g01090.2) possesses additional 23 AAs at the N-terminal compared with SnRK1.1 (At3g01090.1). K48 substitution to M causes a kinase-dead mutant (mSnRK1.1) of SnRK1.1. F, The C-terminal of SnRK1.1 interacts with the C-terminal of SLAH3. The truncated fragments of SnRK1.1 were used to test interactions with the C-terminal of SLAH3 in yeast two-hybrid assay.

Our previous study showed that an anion channel SLAH3 contributes to the process of nitrate-dependent alleviation of ammonium toxicity in Arabidopsis (Zheng et al., 2015). We analyzed the phenotypes of WT Col-0 and the loss-of-function *SLAH3* mutants under the condition of high-ammonium, low-nitrate, and low-pH [5 mM ${\text{NH}}_{4}^{+}$, 1mM ${\text{NO}}_{3}^{-}$ (A5N1), and pH 4.5]. Compared to Col-0, *slah3-3 and slah3-4* mutants showed strong ammonium toxic phenotypes under higher concentrations of ${\text{NH}}_{4}^{+}$ and lower concentrations of ${\text{NO}}_{3}^{-}$. More importantly, elevated pH not only greatly promoted growth in all plants but also eliminated the phenotypic differences between Col-0 and *slah3* mutants. In addition, merely increasing the concentration of ${\text{NO}}_{3}^{-}$ also abolished the ammonium toxic phenotypes of *slah3* mutants (Figure 1A; Zheng et al., 2015). Quantitative analyses performed by measuring the fresh weight were consistent with the phenotypes (Figure 1B). Our results thus indicated nitrate efflux is important for the process of nitrate-dependent alleviation of ammonium toxicity, in which the medium pH plays an essential role. Topologic analyses suggested that SLAH3 has 10 transmembrane helixes with both its NT and CT in the cytoplasm (Figure 1C). The NT of SLAH3, which is around three times larger than the CT, was reported to be important for the regulation of SLAH3 channel activity. For instance, CPK21 phosphorylates the NT of SLAH3 to activate the channel in guard cells (Geiger et al., 2011). However, the reported SLAH3-targeting CPKs show little expression in roots, suggesting additional components are responsible for SLAH3 regulation in roots. To identify more protein kinases involved in SLAH3 regulation, we used a yeast-two-hybrid system to screen the potential interactors of SLAH3. By using the NT and CT of SLAH3 as the baits, we screened the protein collection including all members in the CPK–SnRK superfamily previously constructed by our lab. We identified SnRK1.1 as a potential SLAH3 interactor. Interestingly, unlike the CPKs that interact with the NT of SLAH3, SnRK1.1 showed strong interaction with the CT, suggesting SnRK1.1 functions differently from CPKs (Figure 1D). SnRK1.1 has two isoforms, SnRK1.1-Long (L) and SnRK1.1 (Figure 1E). SnRK1.1-L has additional 23 AA residues at the NT compared to SnRK1.1. SnRK1.1 contains three major domains including kinase, ubiquitin-associated (UBA) and kinase-associated (KA1) domains. The lysine at site 48 (K48) is essential for SnRK1.1 activity and was shown to abolish kinase activity of SnRK1.1 when substituted with methionine (M) (Figure 1E; Cho et al., 2016; Ramon et al., 2019). We next analyzed which domains of SnRK1.1 were responsible for the interaction with SLAH3. Using a yeast-two-hybrid system, we tested the interactions between SLAH3-CT and different truncated SnRK1.1 protein fragments (Figure 1F). Our results indicated the CT of SnRK1.1 including KA1 domain is sufficient to interact with SLAH3-CT. The NT of SnRK1.1 including the kinase domain is not involved in the association with SLAH3-CT. Furthermore, the kinase-dead variant of SnRK1.1 (K48M) showed association with SLAH3-CT, indicating the kinase activity of SnRK1.1 is not required for its interaction with SLAH3-CT (Figure 1F). Thus, our results showed that SnRK1.1, a central regulator in energy signaling and stress responses, interacts with the CT of SLAH3.

### SnRK1.1 interacts with SLAH3 in vivo

**Figure 2:**
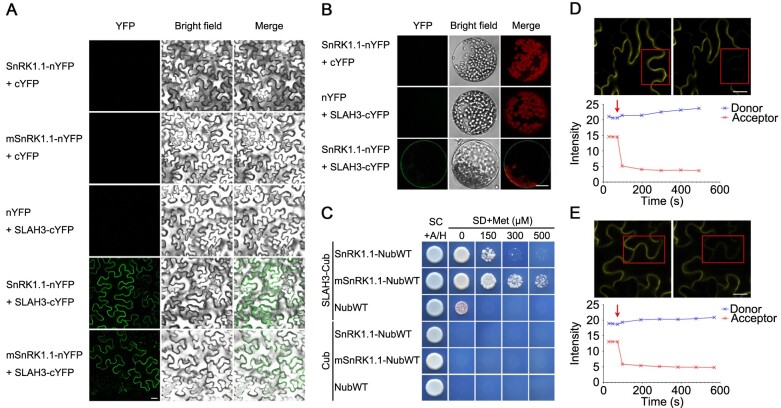
SnRK1.1 interacts with SLAH3 in vivo. A and B, SLAH3 interacts with SnRK1.1 and mSnRK1.1 in leaf cells of *N. benthamiana*. In a BiFC assay, SLAH3 associates with SnRK1.1 and mSnRK1.1 at the plasma membrane in transiently transformed epidermal cells (A) and isolated mesophyll protoplasts (B) of *N. benthamiana*. White scale = 20 µm. C, In the mbSUS assay, full-length SLAH3 interacts with SnRK1.1 and mSnRK1.1. D and E, Acceptor photobleaching method of FRET was used to detect the interaction of SLAH3 with SnRK1.1/mSnRK1.1. The prebleach and postbleach images expressing SLAH3-YFP and SnRK1.1-CFP (D)/mSnRK1.1-CFP (E) were acquired. Region characterized by red box was identified and photobleached. The red arrows represent the beginning of photobleaching. White scale = 20 µm.

To verify the interaction between SnRK1.1 and SLAH3, we utilized bimolecular fluorescence complementation (BiFC) to test the association of SnRK1.1 with full length SLAH3 in vivo. After being co-expressed in the leaf epidermal cells of *Nicotiana benthamiana*, SnRK1.1 interacted with SLAH3 (Figure 2A). Furthermore, the kinase-dead variant (K48M) of SnRK1.1 (mSnRK1.1) still associated with SLAH3, confirming that kinase activity of SnRK1.1 has no effect on the SnRK1.1–SLAH3 interaction. After isolating mesophyll protoplasts of *N. benthamiana* co-expressed with SnRK1.1 and SLAH3, we found SnRK1.1–SLAH3 interaction occurred at the PM (Figure 2B). Next, an assay using the mating-based split ubiquitin system (mbSUS) also confirmed that SnRK1.1 interacted with full length SLAH3 in yeast cells (Figure 2C). Moreover, we used fluorescence resonance energy transfer (FRET) to further verify the interaction between SLAH3 with SnRK1.1 in planta. In the acceptor photobleaching experiment, the images of the same regions before and after photobleaching were acquired to indicate the FRET signal when SnRK1.1/mSnRK1.1-CFP (cyan fluorescent protein) was coexpressed with SLAH3-YFP (yellow fluorescent protein). The fluorescence intensity of the acceptor decreased while that of the donor increased with acceptor bleaching (Figure 2, D and E). In the sensitized emission experiment, the FRET signal was detected when SLAH3-YFP was coexpressed with SnRK1.1/mSnRK1.1-CFP (Supplemental Figure S1). Both of the FRET experiments demonstrated that SLAH3 interacts with SnRK1.1 and mSnRK1.1 in planta (Figure 2, D and E and Supplemental Figure S1).

### *SnRK1.1* and *SLAH3* are expressed in roots

**Figure 3:**
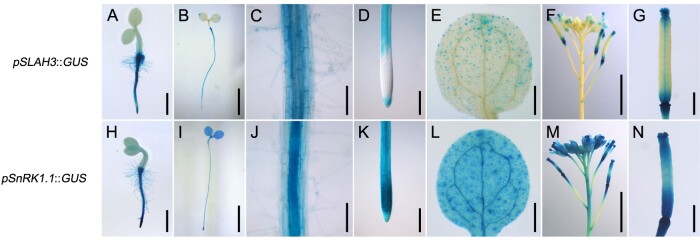
Histochemical analyses of *SLAH3* and *SnRK1.1*. Different developmental stages and different tissues of transgenic plants harboring *pSLAH3::GUS* or *pSnRK1.1::GUS* were collected and stained. A and H, One-day-old seedlings (scale = 1 mm). B and I, Four-day-old seedlings (scale = 5 mm). C and J, The roots of 4-d-old seedlings (scale = 0.1 mm). D and K, The root tips of 4-d-old seedlings (scale = 0.2 mm). E and L, The cotyledons of 4-d-old seedlings (scale = 0.5 mm). F and M, The inflorescences of 5-week-old plants (scale = 5 mm). G and N, The carpels of 5-week-old plants (scale = 1 mm).

We next analyzed whether SnRK1.1 can regulate SLAH3 spatially. The expression patterns of *SnRK1.1* and *SLAH3* were determined by promoter-GUS analyses (Figure 3). *SLAH3* was mainly expressed in root, guard cells, and floral organs. *SnRK1.1* was ubiquitously expressed in the plants and showed high expression in roots, suggesting SnRK1.1 can potentially function as a SLAH3 regulator in roots.

### SnRK1.1 is involved in the process of nitrate-dependent alleviation of ammonium

**Figure 4:**
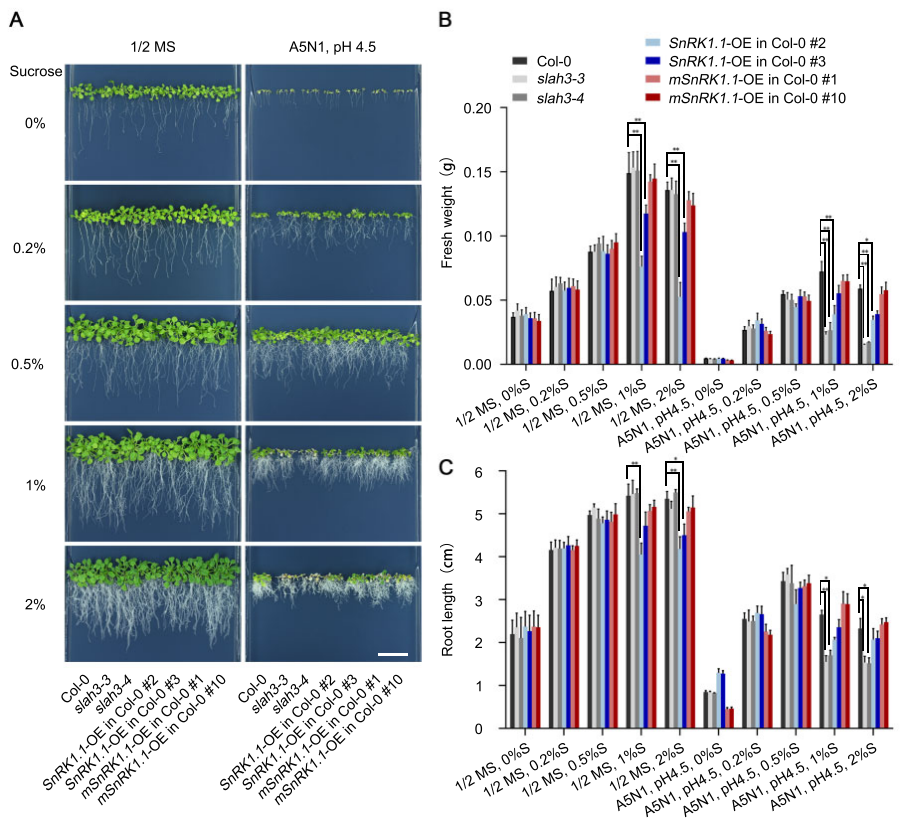
SnRK1.1 functions under high-ammonium/low-pH conditions. A, 20-d-old plants of Col-0, *slah3*, *SnRK1.1*-OE in Col-0 and *mSnRK1.1*-OE in Col-0 are presented under different concentrations of sucrose in 1/2 MS and A5N1, pH 4.5 media. White scale = 2 cm. B, The statistical analyses of fresh weight on the plants present in (A). Fresh weights of 5 seedlings per plate for each line were analyzed. Bars indicate se, *n* = 3. C, The statistical analyses of the root length on the plants present in (A). Root lengths of five seedlings per plate for each line were analyzed. Bars indicate SE, *n* = 3. Statistical analyses were performed by two-way ANOVA analysis with Dunnett’s multiple comparisons test. *P < 0.05; **P < 0.01. S, Sucrose.

To date, two SnRK1s have been found in the Arabidopsis genome, SnRK1.1 and SnRK1.2, which are highly redundant in terms of both structures and functions. The *snrk1.1 snrk1.2* double null mutants are lethal, supporting their essential functions in plants (Baena-González et al., 2007). To further investigate the potential regulation of SLAH3 by SnRK1.1, we generated overexpression lines of *SnRK1.1* and *mSnRK1.1*, driven by a constitutively active *35S* promoter. RT-qPCR results indicated *SnRK1.1* and *mSnRK1.1* were highly expressed in the overexpression plants (Supplemental Figure S2). We analyzed the phenotypes of two independent lines of *SnRK1.1* or *mSnRK1.1* overexpression plants (*SnRK1.1*-OE or *mSnRK1.1*-OE) under the conditions of high-ammonium, low-nitrate, and low-pH (A5N1, pH 4.5), supplemented with different concentrations of sucrose (Figure 4A). The *slah3-3* and *slah3-4* mutant plants, exhibiting no phenotypic differences compared with Col-0 in 1/2 Murashige and Skoog (MS) medium, showed obvious defective phenotypes in A5N1, pH 4.5 medium when supplied with 1% or 2% sucrose. Under same A5N1, pH 4.5 condition, the phenotypic differences between Col-0 and *slah3* mutants were greatly reduced when sucrose was decreased to lower concentrations (0.5%, 0.2%, or 0%). These results indicated the presence of sugars affects the ammonium toxic phenotypes of *slah3* mutants. When A5N1, pH 4.5 conditions was applied with 1% or 2% sucrose, *SnRK1.1*-OE lines and *slah3* mutants showed obvious growth defects compared with Col-0. Under same A5N1, pH 4.5 conditions, the phenotypic differences between *SnRK1.1*-OE lines, *slah3* mutants, and Col-0 were nearly abolished when sucrose was decreased to 0.2%. *SnRK1.1*-OE plants showed inhibited growth in 1/2 MS medium when higher concentrations of sucrose (1% or 2%) were supplemented, but exhibited similar phenotypes to Col-0 when lower concentrations of sucrose (0.5%, 0.2%, or 0%) were applied. Whereas, under A5N1, pH 4.5 condition with no sucrose provided, *SnRK1.1*-OE plants showed even longer root length compared with Col-0. *mSnRK1.1*-OE plants showed inhibited growth in A5N1, pH 4.5 condition with no sucrose supplemented, but exhibited similar phenotypes to Col-0 when sucrose was applied. Quantitative analyses were performed by measuring the fresh weight and root length of the plants (Figure 4, B and C). These results suggested SnRK1.1 plays a role under high-ammonium/low-pH conditions, and sugar is involved in this process. Similar phenotypic characteristics were also observed in the media with different concentrations of glucose, which further indicated that SnRK1 indeed participates in the responses to high-ammonium stress through the regulation of sugar signals (Supplemental Figure S3). In addition, we analyzed the phenotypes in mediums supplemented with different concentrations of palatinose (a nonmetabolizable analog of sucrose) for 20 d. In 1/2 MS mediums, all plants showed no difference when supplemented with palatinose. Under the conditions of A5N1, pH 4.5, compared to wildtype (WT) plants, *SnRK1.1-*OE plants showed enhanced sensitivity to higher concentrations of sucrose but less sensitivity to higher concentrations of palatinose. Meanwhile, supplemented with all concentrations of palatinose, *slah3* mutants showed similar phenotypes to Col-0 (Supplemental Figure S4). Furthermore, the expression of *SnRK1.1* affected the phenotype in a dosage-dependent manner when treated with metabolic sugar (sucrose or glucose). The transgenic lines expressing lower levels of *SnRK1.1* showed weaker phenotype than the lines expressing higher level of *SnRK1.1*. In contrast, the treatment of palatinose had no dosage effect on the phenotype in the plant lines with different *SnRK1.1* expression levels. These results suggested metabolizable sugar such as sucrose or glucose plays a role in SLAH3-mediated nitrate-dependent alleviation of ammonium toxicity. Next, the *SnRK1.1-*OE or *mSnRK1.1*-OE plants were crossed to *slah3-4* background. Under A5N1, pH 4.5 condition, the phenotypes caused by the overexpression of *SnRK1.1* or *mSnRK1.1* were abolished in *slah3-4* background (Supplemental Figure S5, A and C). Statistical analyses were performed by analyzing the fresh weight and root length (Supplemental Figure S5, B and D). These results indicated *SLAH3* is required for *SnRK1.1* in regulating high-ammonium stress adaption. Taken together, these results suggested sugar signaling, in which SnRK1.1 functions as a central regulator, is involved in the process of nitrate-dependent alleviation of ammonium toxicity. SnRK1.1 likely contributes to this process via negatively regulating SLAH3.

### SLAH3 is required for SnRK1.1 to regulate hypocotyl growth in dark

**Figure 5:**
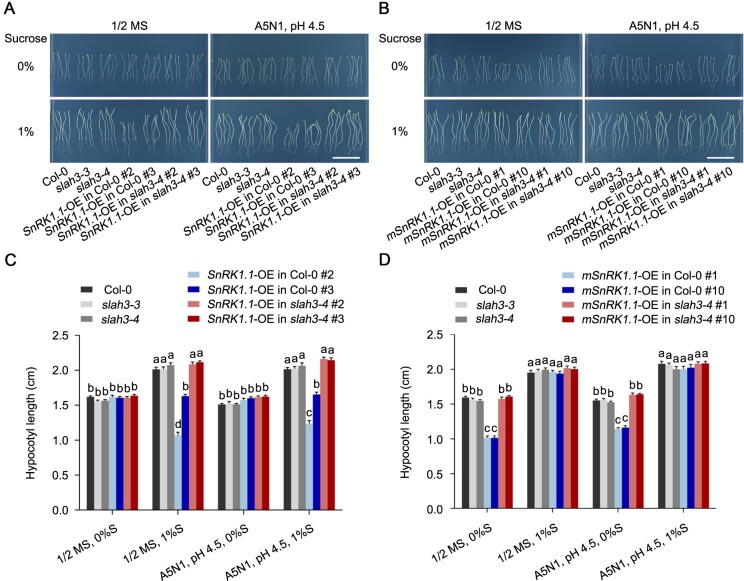
SLAH3 is required for SnRK1.1 to regulate hypocotyl growth in dark. A, The phenotypes of Col-0, *slah3-4*, *SnRK1.1*-OE lines cultivated for 8 d under different culture conditions in dark. White scale = 2 cm. B, The phenotypes of Col-0, *slah3-4*, and *mSnRK1.1*-OE lines cultivated for seven days under different culture conditions in the dark. White scale = 2 cm. C and D, The statistical analyses of hypocotyl length on the plants presented in (A) and (B). Data are shown as means ± se, *n* = 20. Statistical analyses were performed by two-way ANOVA analysis with Tukey’s multiple comparisons test. Significant differences are indicated with different letters. *P* <0.05. S, Sucrose.

SnRK1 kinases play important roles in regulating carbohydrate metabolism and are involved in sucrose-induced hypocotyl elongation of plants in dark (Simon et al., 2018; Hamasaki et al., 2019). Overexpression lines of *SnRK1.1* (*SnRK1.1*-OE) or *mSnRK1.1* (*mSnRK1.1*-OE) in Col-0 or *slah3-4* backgrounds were next analyzed in dark condition. Without sucrose supplement, *SnRK1.1* overexpression caused no phenotypic differences in Col-0 or *slah3-4* under either 1/2 MS or A5N1, pH 4.5 condition. Supplemented with 1% sucrose, however, *SnRK1.1*-OE in Col-0 showed shortened hypocotyl compared with Col-0 in both 1/2 MS and A5N1, pH 4.5 conditions. *SnRK1.1*-OE in Col-0 was crossed to *slah3-4* to generate *SnRK1.1*-OE in *slah3-4*. Interestingly, although *slah3-4* mutant showed similar phenotypes with Col-0 under all conditions tested, the shortened hypocotyl phenotype caused by *SnRK1.1*-OE was suppressed in *slah3-4* (Figure 5, A and C). Different from *SnRK1.1*-OE plants, *mSnRK1.1*-OE lines exhibited no difference with their Col-0 and *slah3-4* backgrounds when 1% sucrose was supplemented, but showed shortened hypocotyl when no sucrose was supplied (Figure 5, B and D). Similarly, the short hypocotyl phenotype caused by *mSnRK1.1* overexpression in Col-0 disappeared in *slah3-4* background on the medium without sucrose. The results surprisingly showed that *SnRK1.1* seems to require *SLAH3* to regulate hypocotyl growth during skotomorphogenesis in response to sugar signals.

### SnRK1.1 phosphorylates SLAH3-CT in vitro

**Figure 6:**
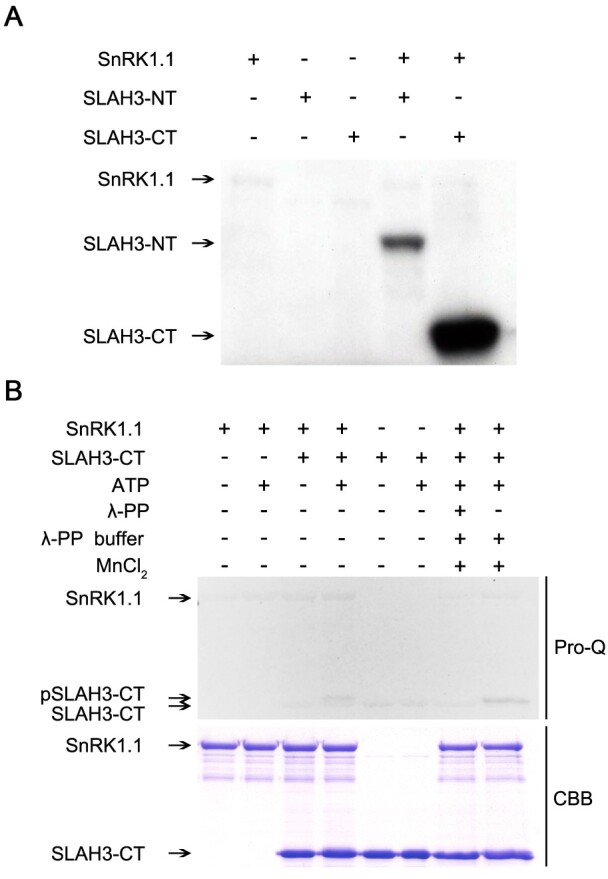
SnRK1.1 phosphorylates the C-terminal of SLAH3. A, SnRK1.1 strongly phosphorylates the C-terminal of SLAH3. The N/C-terminals of SLAH3 and SnRK1.1 were purified in *E. coli* cells. Kinase assay based on ^32^P-labeled ATP was performed followed by autoradiography. B, In vitro kinase assay with Pro-Q staining confirms the phosphorylation of the C-terminal of SLAH3 by SnRK1.1. SnRK1.1 causes band shifting of SLAH3-CT, which is abolished by the treatment of protein phosphatase (λ-PP). CBB staining is presented as loading control.

Given that SnRK1.1 interacts with the CT of SLAH3 and may contribute to SLAH3-mediated physiological process, we next analyzed the detailed molecular mechanism of how SnRK1.1 regulates SLAH3. Kinase assay based on ^32^p-labeled ATP showed SnRK1.1 strongly phosphorylated SLAH3-CT while SLAH3-NT only showed a basal phosphorylation upon SnRK1.1 presence, indicating SnRK1 mainly phosphorylates SLAH3-CT (Figure 6A). The phosphorylation of SLAH3-CT was further confirmed by another kinase assay using a Pro-Q diamond phosphoprotein gel stain by which the phosphorylated proteins on the protein gel can be directly revealed by fluorescence scanner detection. SnRK1.1 caused band shifting of SLAH3-CT in a Pro-Q gel but the shifting band disappeared when protein phosphatase (λ-pp) was added (Figure 6B). Thus, the kinase assays, together with protein interaction results, indicated SnRK1 interacts and strongly phosphorylates SLAH3-CT.

**Figure 7:**
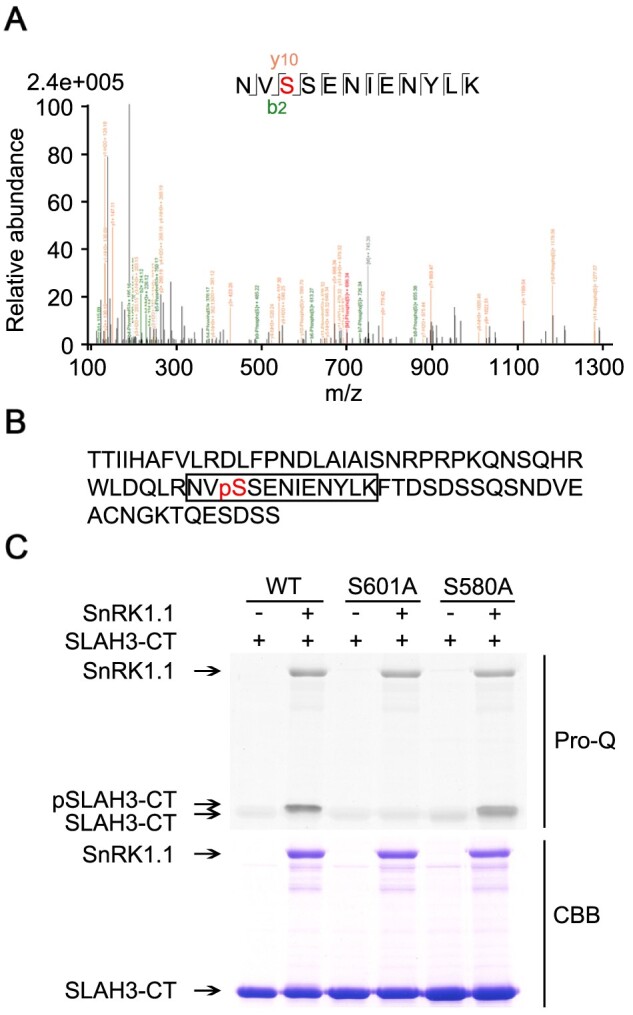
S601 is the only AA residue phosphorylated by SnRK1.1 at the C-terminal of SLAH3. A, LC–MS/MS was used to analyze the phosphorylated site of SLAH3 C-terminal after phosphorylation by SnRK1.1. S601 of SLAH3 was identified as the only site phosphorylated by SnRK1.1. B, The AA sequence of the C-terminal of SLAH3. The detected phosphorylated peptide is framed and the red-colored pS (S601) is the identified phosphorylation site. C, In vitro kinase assay confirms S601 as the sole phosphorylation site of SLAH3-CT by SnRK1.1. S580A substitution is a control. These assays were performed three times and showed similar results.

Liquid chromatograph-mass spectrometer (LC-MS/MS) analysis was next used to determine the specific sites of SLAH3-CT phosphorylated by SnRK1.1 (Figure 7A). The site S601 was identified as the only AA residue of SLAH3-CT phosphorylated by SnRK1.1 (Figure 7B). We then verified the result from MS by using Pro-Q kinase assay. It was previously reported that the T513 of SLAC1 in its CT functions as a crucial phosphorylation site for the activation of SLAC1 (Dreyer et al., 2012; Maierhofer et al., 2014). The conserved serine in SLAH3, S580, was therefore used as a control. When S601 was substituted with A (S601A), the phosphorylation of SLAH3-CT mediated by SnRK1.1 was completely abolished in a Pro-Q kinase assay (Figure 7C). In contrast, S580A substitution did not affect the phosphorylation of SLAH3 by SnRK1.1. Thus, the biochemical and MS assays revealed that SnRK1.1 phosphorylates S601 at the CT of SLAH3.

### Phosphorylation of S601 of SLAH3 by SnRK1.1 plays an important role in high-ammonium response

**Figure 8:**
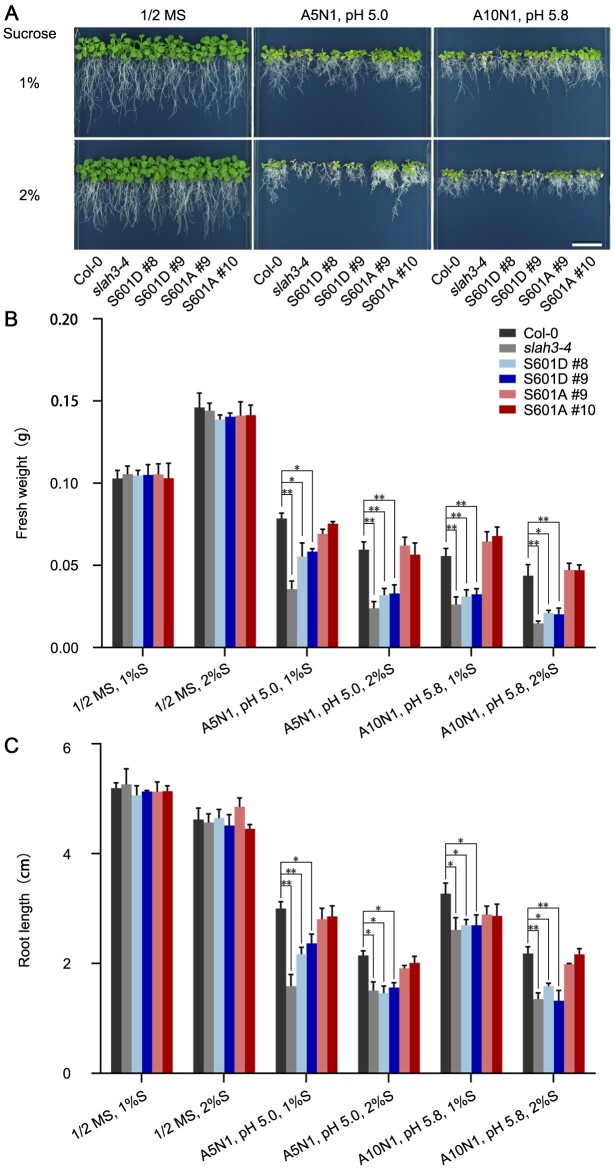
S601 of SLAH3 plays a role in regulating nitrate-dependent alleviation of ammonium toxicity. A, 20-d-old plants of Col-0, *slah3-4* and *slah3-4* complemented with different variants of *SLAH3* sequences are presented under different culture conditions. The phospho-dead variant (*S601A*) and phospho-mimetic variant (*S601D*) were driven by its native promoter. White scale = 2 cm. B, The statistical analyses of fresh weight on the plants presented in (A). Fresh weights of five seedlings per plate for each line were analyzed. Bars indicate se, *n* = 3. C, The statistical analyses of main root length on the plants presented in (A). Root lengths of five seedlings per plate for each line were analyzed. Bars indicate se, *n* = 3. Statistical analyses were performed by two-way ANOVA analysis with Dunnett’s multiple comparisons test. *P < 0.05; **P < 0.01. S, Sucrose.

Since S601 of SLAH3 was phosphorylated by SnRK1.1 in the in vitro assays, we next verified the importance of S601 for SLAH3 function. To understand the biological role of S601, we generated transgenic plants harboring SLAH3 phosphorylation-mimetic (*S601D*) or phosphorylation-dead (*S601A*) mutation driven by its native promoter in the *slah3-4* background. Reverse transcription-polymerase chain reaction (RT-PCR) indicated the transcripts of *SLAH3 S601D* or *S601A* in complemented lines showed similar levels as those in Col-0 (Supplemental Figure S6). *S601A* mutation fully rescued the phenotypes of *slah3-4* under A5N1 condition. In contrast, *S601D* mutation can barely complement the phenotypes of *slah3-4* under A5N1 condition (Figure 8A). Quantitative analyses were performed by measuring the fresh weights and root lengths of the plants (Figure 8, B and C). Yeast-two-hybrid assay showed *S601A* or *S601D* mutation did not affect the interaction between SLAH3-CT and SnRK1.1 (Supplemental Figure S7). In contrast, SLAH3 completely complemented the phenotypes of *slah3-4* under A5N1 condition (Supplemental Figure S8). The genetic results, therefore, supported the notion that S601 phosphorylation mediated by SnRK1.1 may negatively regulate SLAH3 function in regulating the process of nitrate-dependent alleviation of ammonium toxicity.

**Figure 9:**
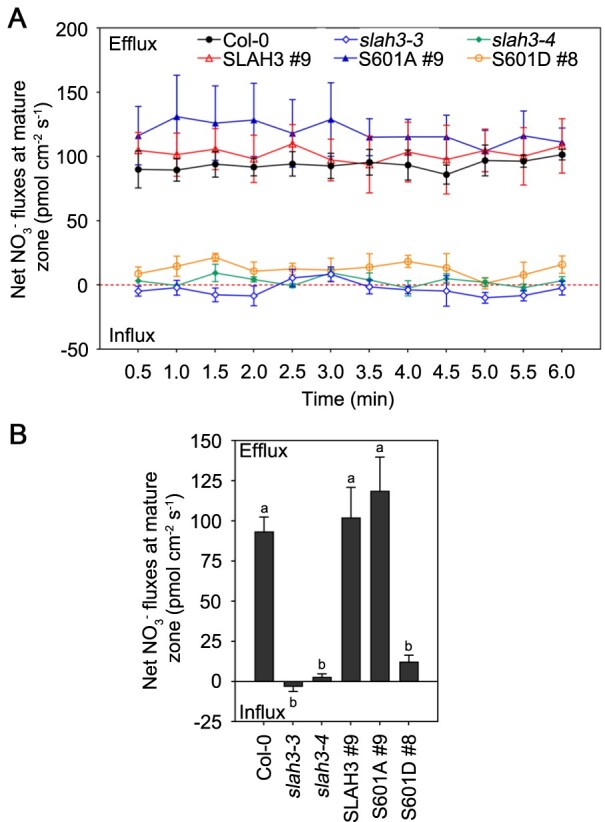
SLAH3 S601 influences the net ${\text{NO}}_{3}^{-}$ fluxes at the maturation zone in primary roots under high-ammonium/low-pH condition. A, The net ${\text{NO}}_{3}^{-}$ fluxes in Col-0, *slah3-3*, *slah3-4*, and complementary lines seedlings that were grown on 1/2 MS medium for eight days and transferred to high-${\text{NH}}_{4}^{+}$ conditions (1 mM ${\text{NO}}_{3}^{-}$, 10 mM ${\text{NH}}_{4}^{+}$, pH 4.5) for 2 h were measured by NMT. The ${\text{NO}}_{3}^{-}$ fluxes at the maturation zone next to root tip in primary roots of Arabidopsis were measured. Each point represents the mean ± se of three individual plants. B, Mean values of ${\text{NO}}_{3}^{-}$ fluxes from (A) are presented. Data are shown as means ± se, *n* = 3. Statistical analyses were performed by one-way ANOVA with Tukey’s multiple comparisons test. Significant differences among all plants are indicated with different letters. *P* <0.05.

To further understand the physiological role of the SLAH3 S601 site, we analyzed the nitrate fluxes by using non-invasive micro-test technology (NMT) method. To test whether Cl^−^interferes with the measurements of ${\text{NO}}_{3}^{-}$ in the NMT system, the ${\text{NO}}_{3}^{-}$ concentrations and net fluxes in measuring solution 1 (1 mM KNO_3_, 0.1 mM KCl, 0.1 mM CaCl_2_, 0.3 mM MES, pH 6.0) and measuring solution 2 (1 mM KNO_3_, 1.1 mM KCl, 0.1 mM CaCl_2_, 0.3 mM MES, pH 6.0) were detected without seedlings. The presence of Cl^−^ did not interfere with the measurements of ${\text{NO}}_{3}^{-}$ (Supplemental Figure S9). Next, we detected the net ${\text{NO}}_{3}^{-}$ fluxes at the maturation zone next to the primary root tip (Figure 9A, Supplemental Figure S10A). Seedlings from Col-0, *slah3-3*, *slah3-4*, and all complementary lines that were grown on 1/2 MS medium for 8 d and transferred to high-${\text{NH}}_{4}^{+}$/low-pH condition (1 mM ${\text{NO}}_{3}^{-}$, 10 mM ${\text{NH}}_{4}^{+}$, pH 4.5) or non-high-${\text{NH}}_{4}^{+}$/low-pH condition (1 mM ${\text{NO}}_{3}^{-}$, 1 mM ${\text{NH}}_{4}^{+}$, pH 5.7) for 2 h were measured. ${\text{NO}}_{3}^{-}$ fluxes from the plant roots were detected over 6 min (Figure 9B;  Supplemental Figure S10B). Under high-${\text{NH}}_{4}^{+}$/low-pH stress, the mean value of ${\text{NO}}_{3}^{-}$ effluxes from Col-0 was around 93 pmol cm^−2^ s^−1^, and the mean value of ${\text{NO}}_{3}^{-}$ effluxes from *slah3* mutants was around 0 pmol cm^−2^ s^−1^. Complementary lines of *SLAH3* and *SLAH3 S601A* in *slah3-4* background showed nitrate effluxes similar to Col-0 plants. In contrast, complementary lines of *SLAH3 S601D* in *slah3-4* background showed nitrate effluxes similar to *slah3* mutants (Figure 9B). In addition, under non-high-${\text{NH}}_{4}^{+}$/low-pH stress, nitrate effluxes were similar among all plants, and the mean value of ${\text{NO}}_{3}^{-}$ effluxes were around 0 pmol cm^−2^ s^−1^ (Supplemental Figure S10). These results suggested that S601 of SLAH3 plays an important role in regulating nitrate efflux under high-${\text{NH}}_{4}^{+}$/low-pH stress.

### High-ammonium stress causes accumulation of SnRK1.1 in nucleus

**Figure 10:**
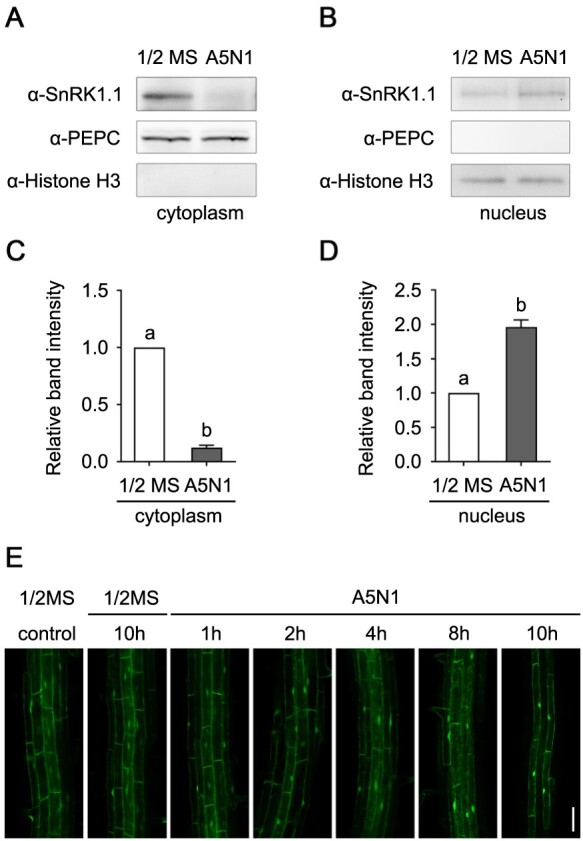
SnRK1.1 transfers from cytoplasm to nucleus under high-ammonium/low-pH conditions. A and B, The abundance of SnRK1.1 protein is decreased in cytoplasm (A) and increased in nucleus (B) under high-ammonium/low-pH condition. In Col-0 plants grown for 14 d in 1/2 MS and A5N1, pH 4.5 conditions, the protein contents in cytoplasm and nucleus were separated. SnRK1.1 protein abundance was analyzed by immunoblotting using an anti-SnRK1.1 antibody. Anti-PEPC and anti-Histone H3 antibodies were used to detect PEPC and Histone H3, marker proteins for cytoplasmic and nuclear sections, respectively. These assays were performed three times and showed similar results. C and D, The quantitative values of the band intensities of SnRK1.1. The ratios of SnRK1.1 to PEPC (C) and Histone H3 (D) were calculated. Data are shown as means ± se, *n* = 3. Statistical analyses were performed by one-way ANOVA with Tukey’s multiple comparisons test. Significant differences are indicated with different letters. *P* <0.05. E, Transient treatment of high-ammonium/low-pH conditions causes accumulation of SnRK1.1 in nucleus. The Col-0 plants expressing YFP-SnRK1.1 grown for 5 d in 1/2 MS were transferred to liquid 1/2 MS or liquid A5N1, pH 4.5 condition. Starting from 2 h, in the plants that were subjected to A5N1 condition, YFP-SnRK1.1 signal was clearly detected to be accumulated in nucleus. White scale = 50 μm.

Under normal condition, SnRK1.1 locates in both cytoplasm and nucleus. A recent study demonstrated that cytosol-localized SnRK1.1 migrates to nucleus under metabolic, hypoxia, DCMU and dark stresses (Ramon et al., 2019). Therefore, we tested whether SnRK1.1 also accumulates in the nucleus under high-ammonium stress. We analyzed the distribution of SnRK1.1 protein upon high-ammonium treatments. In the Col-0 plants grown on 1/2 MS or A5N1, pH 4.5 medium with 0.9% agarose culture condition for 14 d, the cellular contents in cytoplasm and nucleus were separated. An anti-SnRK1.1 antibody was used to immunoblot SnRK1.1 protein in cytoplasmic or nuclear fraction. Anti-PEPC and anti-Histone H3 antibodies were used for positive indicators for cytoplasmic and nuclear sections, respectively. SnRK1.1 was detected in both cytoplasm and nucleus when the plants were grown in 1/2 MS (Figure 10, A and B). In contrast, when cultivated in A5N1, pH 4.5 media, the abundance of SnRK1.1 protein significantly decreased in cytoplasm but increased in nucleus accordingly (Figure 10, A and B). Quantitative analyses were performed by measuring the relative intensity of SnRK1.1 bands (Figure 10, C and D). The images of original western blots are included in supplementary data (Supplemental Figure S11). Furthermore, we analyzed the responses of SnRK1.1 protein to short-term high-ammonium stress. The Col-0 plants expressing YFP-SnRK1.1 were grown in the 1/2 MS medium for 5 d. The transgenic plants were transferred to the liquid 1/2 MS medium or liquid A5N1, pH 4.5 medium, and the YFP signal was checked at different time courses. In the control plants that were transferred to the liquid 1/2 MS medium, the distribution of SnRK1.1 was not altered and was detected in both cytoplasm and nucleus. In contrast, in the plants subjected to A5N1, pH 4.5 condition, the SnRK1.1 protein gradually accumulated in nucleus. Within 10 h, most SnRK1.1 protein was detected in nucleus under A5N1, pH 4.5 conditions (Figure 10E). Our results indicated SnRK1.1 accumulates in nucleus under both long-term and short-term high-ammonium stress conditions.

## Discussion

**Figure 11:**
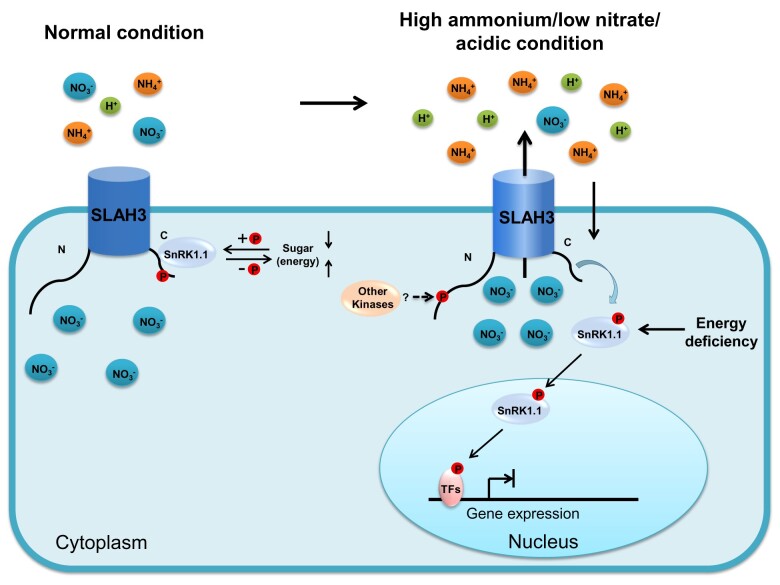
Hypothetical model for regulation of SLAH3 by SnRK1.1. Under normal condition, cytosol-localized SnRK1.1 interacts and phosphorylates the C-terminal of SLAH3 at S601 to inhibit SLAH3 activity. The negative regulation on SLAH3 by SnRK1.1 prevents nitrate loss. The activity of SnRK1.1 is regulated by energy homeostasis. In response to high-ammonium/low-pH stress, SnRK1.1 migrates to nucleus, which releases the inhibition on SLAH3 and leads to nitrate efflux. The accumulated nitrate in the rhizosphere contributes to the alleviation of ammonium toxicity. Severe energy deficiency strongly activates nucleus-localized SnRK1.1 that promotes the phosphorylation of the transcription factors regulating the expression of responsive genes.

SnRK1.1 acts as a central regulator responsible for multiple stress-related signaling. In this study, SnRK1.1 is identified as an important component contributing to nitrate-dependent alleviation of ammonium toxicity via negatively regulating SLAH3. In our hypothetical model, relatively lower levels of sugar lead to activation of SnRK1.1, likely in the sink organs such as root. SnRK1.1 is localized to both cytoplasm and nucleus under normal condition. Upon activation, the cytosol-localized SnRK1.1 phosphorylates SLAH3-CT at S601, leading to SLAH3 inhibition. Under normal condition, the activation of SnRK1.1 is regulated by cellular energy status. The negative regulation of SLAH3 by SnRK1.1 may serve as a strategy to reduce nitrate loss in root under normal condition. It is likely an important mechanism of how plants coordinate energy homeostasis and nutrient usage. When the plants are subjected to high-ammonium stress, cytosol-localized SnRK1.1 migrates to nucleus. The translocation of SnRK1.1 releases its inhibitory effect on SLAH3, which in turn allows SLAH3 activation mediated by an unknown kinase(s). At the same time, high-ammonium/low-pH stress condition may cause severe energy deficiency, which leads to strong activation of nucleus-localized SnRK1.1 that regulates downstream components such as transcription factors responsible for the expression of energy/stress-related genes. Nitrate efflux mediated by SLAH3 ultimately causes nitrate accumulation in rhizosphere, which is sufficient to detoxify high-ammonium condition (Figure 11). Unlike the previously identified SLAH3 regulators that phosphorylate the NT of SLAH3 and function as SLAH3 activators, our study revealed that stress-related protein kinase SnRK1.1 phosphorylates the CT of SLAH3 and potentially acts as a SLAH3 inhibitor.

It is puzzling that for many plants ${\text{NH}}_{4}^{+}$ is toxic N source when it is dominant over ${\text{NO}}_{3}^{-}$ because ${\text{NH}}_{4}^{+}$ can be directly assimilated after absorption into the cell (Britto et al., 2001; Britto and Kronzucker, 2002). ${\text{NO}}_{3}^{-}$ needs to be reduced into ${\text{NH}}_{4}^{+}$ via a two-step process, which is energy-consuming, before it can be utilized by plants (Crawford, 1995; Xu et al., 2012). In contrast to ${\text{NH}}_{4}^{+}$, ${\text{NO}}_{3}^{-}$ usually does not cause obvious toxicity in plants when it is the sole N nutrient. The detailed mechanisms how a theoretically preferred nitrogen source inhibits plant growth are still largely unclear. The proposed causes of ammonium toxicity include futile ammonium cycling, inorganic cation depletion, organic acid depletion, accumulation of ROS, and medium acidification (Britto et al., 2001; Koyama et al., 2001; Britto and Kronzucker, 2002). However, no sufficient evidence is provided to clearly describe these mechanisms so far. Small amounts of nitrate can efficiently detoxify ammonium with unknown mechanisms (Domene and Ayres, 2001; Britto and Kronzucker, 2002; Houdusse et al., 2005). Our previous study revealed that a nitrate efflux channel, SLAH3, contributes to nitrate-dependent ammonium detoxification (Zheng et al., 2015). SLAH3-mediated nitrate efflux contributes to nitrate-dependent alleviation of ammonium toxicity, in which medium pH is an essential factor.

*SLAC1* was the first characterized gene in the *SLAC*/*SLAH* family. SLAC1 regulates stomatal movement upon ABA signal or PAMP stimulus through Ca^2+^-dependent and Ca^2+^-independent pathways (Brandt et al., 2012; Maierhofer et al., 2014; Zheng et al., 2018). *SLAH3* functions redundantly with *SLAC1* in guard cells (Negi et al., 2008; Geiger et al., 2011; Demir et al., 2013). Nevertheless, distinct from *SLAC1* that is restricted to guard cells, *SLAH3* is expressed ubiquitously in pant tissues such as root and pollen tube, suggesting additional functions of SLAH3 besides controlling stomatal movement (Gutermuth et al., 2013; Zheng et al., 2015). Protein kinases were identified to phosphorylate SLAH3 to activate the SLAH3 channel. CPK21 activates SLAH3, a component of nanodomain in mesophyll cells, which is regulated by an ABA-signaling mediator, phosphatase ABI1 (Geiger et al., 2011; Demir et al., 2013). SLAH3 also functions in pollen tube tip likely regulating anion homeostasis during pollen tube growth (Gutermuth et al., 2013). Earlier studies have shown that the NT phosphorylation of SLAH3 or SLAC1 is involved in channel activation mediated by CPKs, SnRK2s, and CIPKs (Geiger et al., 2009; Maierhofer et al., 2014; Kudla et al., 2018). CPK21 interacts with the NT of SLAH3 and gates the opening of SLAH3. Therefore, the phosphorylation of NT of SLAH3 serves as a positive control for SLAH3 channel activity. A recent report showed an RLCK, PBL27, also phosphorylates the NT and opens SLAH3. However, whether the CT phosphorylation of SLAH3 also regulates SLAH3 activity is still unclear (Liu et al., 2019). Despite the discoveries that the CT of SLAH3 can be phosphorylated by different protein kinases, the physiological roles of the phosphorylation of SLAH3-CT are still beyond our understandings. In this study, we demonstrate that SnRK1.1 kinase can strongly phosphorylate the CT of SLAH3. More importantly, by using a combination of biochemical, molecular, genetic and NMT analyses, we show that this SnRK1.1-mediated CT phosphorylation likely serves as a negative regulation on SLAH3 (Figures 8 and 9; Supplemental Figure S8). Our study thus provides additional insights into the regulatory mechanisms of SLAH3.

SnRK1/SNF1/AMPK protein kinases are proposed to function as essential energy sensors in regulating energy homeostasis in eukaryotes (Polge and Thomas, 2007; Crepin and Rolland, 2019). SnRK1/SNF1/AMPK function as heterotrimeric complexes consisting of three subunits consisting of a catalytic α-subunit, a scaffold β-subunit and an adenine nucleotide AMP or ADP-associating γ subunit (Polge and Thomas, 2007; Crozet et al., 2014; Crepin and Rolland, 2019). In plants, SnRK1 complex contains α kinase subunit SnRK1.1 or SnRK1.2, a plant-specific β subunit and a unique hybrid βγ subunit which lacks AMP binding domain (Broeckx et al., 2016; Crepin and Rolland, 2019). Different from SNF1 and AMPK that require adenine nucleotide-binding to be activated, SnRK1 α subunit possesses auto-phosphorylation in a complex-independent manner (Broeckx et al., 2016; Crepin and Rolland, 2019). The activation of SnRK1, distinct from SNF1 and AMPK, involves the removal of repressors but not association with adenine nucleotides (Crozet et al., 2014; Maya-Bernal et al., 2017; Emanuelle et al., 2018; Ramon et al., 2019). Intriguingly, this negative regulation of SnRK1.1 seems to be in line with its negative role in regulating SLAH3. Negative regulations seem to be plausible in plants, which often cause rapid downstream responses (Crepin and Rolland, 2019).

In Xenopus oocytes, SLAH3 is silent without activators and extracellular ${\text{NO}}_{3}^{-}$ (Geiger et al., 2011). However, in the natural conditions, the activators of SLAH3 can always be dynamically activated and there is always nitrate present. Therefore, SLAH3 can be activated and its activity needs to be repressed to prevent nitrate loss under normal conditions. In our model, the repression of SLAH3 activity can be released upon stressed conditions. Under higher concentrations of extracellular ${\text{NO}}_{3}^{-}$, the activity of SLAH3 should be increased (Geiger et al., 2011). However, we believe that extracellular ${\text{NO}}_{3}^{-}$-mediated SLAH3 activation barely contributes to nitrate-dependent alleviation of ammonium toxicity. Under high-ammonium/low-nitrate condition (A5N1, pH 4.5), *slah3* mutants showed severe ammonium toxic phenotype compared to WT (Figure 1A). Whereas, when the concentration of nitrate was increased (A5N5, pH 4.5), the phenotypic difference between *slah3* mutants and WT disappeared (Figure 1A). These results indicated SLAH3-mediated ammonium detoxification occurs under low concentrations of nitrate. Given that higher extracellular ${\text{NO}}_{3}^{-}$ will cause stronger SLAH3 activity, the result that SLAH3 can only detoxify ammonium under low extracellular ${\text{NO}}_{3}^{-}$ demonstrated that nitrate-activated SLAH3 activation unlikely contributes to SLAH3-mediated nitrate-dependent alleviation of ammonium toxicity. Thus, we speculated that the activation and inactivation of SLAH3 by intracellular regulators including unknown kinases and SnRK1.1, instead of extracellular ${\text{NO}}_{3}^{-}$, play a more important role in SLAH3-mediated nitrate-dependent alleviation of ammonium toxicity. Even though nitrate alleviation of ammonium toxicity could be in part resulting from the activation of SLAH3 by nitrate itself, it plays much less of a role in this process.

The activities of channels and transporters are fine-tuned by regulators such as kinases and phosphatases in order to fulfill physiological activities and to respond to various stress conditions (Köhler and Blatt, 2002; Geiger et al., 2009; Hedrich, 2012). Interesting enough, a number of the members in the CPK-SnRK superfamily are engaged in these regulations (Hedrich, 2012). CPK–SnRK superfamily mainly contains CPKs family and SnRK family that comprises SnRK1, SnRK2, and SnRK3 subfamilies (Hrabak et al., 2003). Numerous reports have shown that CPKs, SnRK2s, and SnRK3s are extensively involved in phosphorylating and regulating channels and transporters (Kudla et al., 2018). Despite that SnRK1s have overlapping substrate specificity with CPKs and SnRK2/3, they have not been shown to act as channel or transporter regulators. Our study indicated SnRK1.1, like other members in the CPK–SnRK superfamily, also functions in regulating ion channel via phosphorylation.

Besides its role in sugar signaling, SnRK1.1 is also suggested to mediate multiple stress responses (Baena-González and Sheen, 2008; Hulsmans et al., 2016). The functional analyses of SnRK1.1 demonstrate a complicated network in which a variety of stress signaling is connected by SnRK1.1. Our results suggested that high-ammonium/low-pH stress links with other stress response pathways, in which SnRK1 may function as a key regulator in the crosstalk.

A recent study revealed that PBL27 phosphorylates both NT (S127 and S189) and CT (S601) of SLAH3 during PAMP-induced stomatal closure (Liu et al., 2019). PBL27 can phosphorylate S127 and S189 to activate SLAH3. S601A or S601D did not affect SLAH3 activation by PBL27. However, when SLAH3 S127D (or S189D)/S601D double mutation and PBL27 were coexpressed in oocyte, the SLAH3 S127D (or S189D)/S601D activation seemed to be reduced compared to coexpression of WT SLAH3, S127D, S601D, or S189D with PBL27 (Liu et al., 2019). These results indicated that although S601D single mutation does not affect SLAH3 activity, S601D may function together with other phosphorylation sites to regulate SLAH3 activity. Interestingly, based on their results, S601D may negatively regulate SLAH3 activation which is consistent with our results. Taken together, we conjecture that SLAH3 may be activated via N-terminal phosphorylation but be inhibited by S601 phosphorylation at C-terminal mediated by SnRK1.1. However, the activators that regulate SLAH3 in the process of nitrate-dependent alleviation of ammonium toxicity have not been identified yet. Whether S601 phosphorylation directly regulates the channel activity of SLAH3 remains to be clarified further. In addition, it is also possible that S601 phosphorylation leads to recruiting additional regulatory components, which are directly involved in regulating SLAH3 channel activity.

## Materials and methods

Primer sequences used for all experiments are available in the Supplemental Table 1.

### Plant materials and growth conditions

The *slah3-3* (SALK_106054) and *slah3-4* (SALK_111623) T-DNA insertion alleles were obtained from the ABRC. Overexpression plants were in the Columbia (Col-0) background. The overexpression plants were crossed to *slah3-4* to create the overexpression lines in *slah3-4* background. All complementary lines were in *slah3-4* background.

Arabidopsis (*Arabidopsis thaliana*) grown in soil were cultivated in a green house at 22°C under a 16-h-light/8-h-dark photoperiod. Sterilized plump seeds were vernalized in the dark at 4°C for 3 d and sowed on plates. 1/2 MS medium (Phytotech) and Nitrogen-free MS medium (Caisson) supplemented with different concentrations of ammonium and nitrate were used. Plates were sealed and incubated in growth chambers at 22°C under a 16-h-light/8-h-dark photoperiod. For phenotypic analyses, and extraction and fractionation of proteins from nucleus and cytoplasm, 0.9% (w/v) agarose was added to the media. In the experiments of GUS staining, RNA isolation, subcellular localization, and measurement of ${\text{NO}}_{3}^{-}$ fluxes, 0.9% (w/v) agar was added to the media.

### Plasmid construction

The Gateway gene cloning system (Invitrogen) was used to generate the plasmids used in most experiments. PCR-amplified sequences were cloned into the pDONR by Gateway BP reaction, followed by Gateway LR reaction to construct destination vectors. For constructing overexpression plants, target genes were cloned into binary vector pBIB-BASTA-35S-GWR. For promoter-GUS constructs, the promoter sequences about 1.6 kb were cloned into binary vector pBIB-BASTA-GWR-GUS. For constructing complementary plants, *SLAH3* CDSs with the stop codon were cloned into binary vector pBIB-BASTA-pSLAH3-GWR. For BiFC assay, the coding sequences of target genes were cloned into vector pEG202-GWR-nYFP or pEG202-GWR-cYFP. For constructs used in the yeast two-hybrid assays, the target gene fragments were cloned in vector pGBT9BS-GWR or pGADGH-GWR. For mbSUS assay, the CDSs were cloned in pMetYCgate and pX-NubWTgate plasmids. For prokaryotic expression, the target gene fragments were cloned into the vector pDEST15-GST-GWR. Site-directed mutagenesis was performed with PCR using complementary mutation primer pairs extending 15 nucleotides on either side of the modification. DpnI was used to digest the methylated template DNA. For YFP signal detection, the coding sequences of target genes were cloned into vector pEarleyGate-BASTA-35S-YFP-GWR. For CFP signal detection, the coding sequence of target gene was cloned into vector pBIB-BASTA-35S- GWR-CFP.

### Gene expression analysis

For quantification of gene expression in seedlings, total RNA extraction was performed with RNAprep pure Plant Kit (TIANGEN) according to the manufacturer’s instructions. Two micrograms of total RNA were used for reverse transcription with the Reverse Transcription System (Takara). *ACTIN2* (*ACT2*) was used as a reference gene. RT-qPCR was performed according to the manufacturer’s instructions. The PCR program was applied by a StepOne Plus Real Time PCR system (Applied Biosystems). The expression of *ACTIN2* was used as an internal control to normalize all data. Each experiment was performed using at least three independent biological replicates.

### GUS staining

Different developmental stages and different tissues of transgenic plants were collected and stained. Tissues were first incubated in rinse solution (34.2 mM Na_2_HPO_4_, 15.8 mM NaH_2_PO_4_, 0.5 mM K_3_Fe(CN)_6_, and 0.5 mM K_4_Fe(CN)_6_.3H_2_O) for 5 min, then incubated in stain solution (34.2 mM Na_2_HPO_4_, 15.8 mM NaH_2_PO_4_, 0.5 mM K_3_Fe(CN)_6_, 0.5 mM K_4_Fe(CN)_6_.3H_2_O, and 2 mM X-Gluc) at 37°C for appropriate time. After GUS staining, the plant tissues were immersed in 30%, 50%, 75%, and 95% ethanol for 1 h in turn, and then immersed in 75% ethanol. After being decolorized, the tissues were observed with the Stereomicroscope (Leica).

### Yeast two-hybrid assays

The Yeastmaker Transformation System 2 (Clontech) was used according to the manufacturer’s manual. The interaction tests were performed in yeast (*Saccharomyces cerevisiae*) strain AH109 (Clontech). To identify SLAH3-interacting proteins, the N-terminal and C-terminal region of SLAH3 coding sequences were cloned into pGBT9BS as the bait. cDNAs of the genes in *CPK-SnRK* superfamily were cloned from into pGADGH as prey. For analyzing the interaction between SnRK1.1 and C-terminal of SLAH3, the full-length coding sequence and various truncated coding regions of SnRK1.1 were cloned into pGADGH. Empty vector combinations were used as negative controls.mbSUS was performed as previously described (Obrdlik et al., 2004). The coding sequence of SnRK1.1 was cloned into pX-NubWTgate vector to transform yeast strain THY.AP5, and the full-length coding sequence of SLAH3 was cloned into pMetYCgate vector to transform yeast strain THY.AP4. The empty vectors were used as negative control. Interactions were examined by growing diploid yeast cells on a synthetic dextrose (SD) minimal medium containing 0, 150, 300, or 500 μM Met.

### BiFC and subcellular localization assay

BiFC assay was performed as previously described (Cui et al., 2018). For isolating leaf mesophyll protoplast, 1.5 cm diameter infiltrated leaves were treated with d-Mannitol (500 mM) for 30–60 min, then incubated with 500 µL protoplast solution containing 500 mM d-Mannitol, 10 mM CaCl_2_, 5 mM MES (pH 5.6), 3% (w/v) Cellulase-Onozuka, and 0.75% (w/v) Macerozyme in vacuum for 5 min. The samples were slightly vibrated in the darkness for 4 h, then transferred to the tubes and added half volume of CaCl_2_ (200 mM). The materials were centrifuged at 40*g* for 5 min. The precipitates resuspended with 1 mL Mannitol (500 mM) and 1 mL CaCl_2_ (200 mM) were centrifuged at 40*g* for 5 min. The protoplasts were resuspended with 200–500 µL W5 solution (125 mM CaCl_2,_ 5 mM KCl, 154 mM NaCl, 5 mM glucose, 1.5 mM MES, adjust pH 5.6 with KOH). Leaf mesophyll protoplasts were observed using a two-photon laser confocal microscope (Olympus FV1000 MPE). For YFP visualization, excitation at 514-nm and detection between 525-nm and 620-nm were used. Laser power and PMT gain were adjusted appropriately. Excitation and emission settings of all samples were kept constant. Images were analyzed with image FV10-ASW software.

Subcellular localization of SnRK1.1 was determined by observing the YFP fluorescence signal. The *YFP-SnRK1.1* overexpression plants were grown on 1/2 MS medium for 5 d, and then transferred to 1/2 MS or A5N1, pH 4.5 liquid medium, respectively. The YFP fluorescence signal was observed by using a laser scanning confocal microscope (Nikon A1R+ Ti2-E). For YFP visualization, excitation at 514-nm and detection between 525-nm and 620-nm were used. Laser power and PMT gain were adjusted appropriately. Excitation and emission settings of all samples were kept constant.

### FRET assay

To perform sensitized emission method, a set of three filter configurations were preset and three samples were prepared: one containing donor only (SnRK1.1/mSnRK1.1-CFP), one containing acceptor only (SLAH3-YFP), and one containing both donor and acceptor. Each of the samples was imaged with a donor filter set, a FRET filter set, and an acceptor filter set. The FRET filter set was configured to have an exciter that matched with the donor filter set and an emitter filter that matched with the acceptor filter set. To visualize emission, the three tracks were analogous to a donor (CFP) filter set (track 1: laser, 458-nm; emission 463–510 nm), a FRET filter set (track 2: laser, 458-nm; emission 520–620 nm), and an acceptor (YFP) filter set (track 3: laser, 514-nm; emission 520–620 nm). Laser power and PMT gain were adjusted appropriately. Excitation and emission settings of three samples were kept constant. Images from each track were displayed in separate image channels.

To perform acceptor photobleaching method, regions of interest were chosen and bleaching parameters were defined, and then the predetermined regions were photobleached. The prebleach and postbleach images were acquired. The fluorescence intensity was recorded in each region before, during, and after selectively photobleaching. All FRET experiments were performed using the ZEN 3.1 system combined with a Zeiss LSM 880 confocal microscope.

### Extraction and fractionation of proteins from nucleus and cytoplasm

Arabidopsis Col-0 seeds were cultivated on 1/2 MS and A5N1, pH 4.5 media with 0.9% agarose for 2 weeks, respectively. The seedlings were immediately frozen and ground well in liquid nitrogen. Cells were lysed with 3-mL lysis buffer containing 10 mM Tris–HCl (pH 7.5), 10 mM NaCl, 10 mM MgCl_2_, 10% (v/v) Glycerol, 10 mM β-mercaptoethanol, and 0.1% protease inhibitor cocktail (Roche). Lysates were then filtered twice through a 100-mesh filter membrane. The filtrates were centrifuged at 2,500*g* for 10 min at 4°C. The supernatants were cytoplasmic crude extracts and the precipitates were nuclear crude extracts. The supernatants were transferred to new tubes and centrifuged at 13,000*g* for 15 min at 4°C. The supernatants were cytoplasmic fractions. The nuclear crude extracts were resuspended in 20 mL nuclear resuspension buffer containing 10 mM Tris–HCl (pH 7.5), 10 mM NaCl, 10 mM MgCl_2_, 10 mM β-mercaptoethanol, 1 M hexylene glycol, 0.5% (v/v) Triton X-100, and 1 mM protease inhibitor cocktail, centrifuged at 1,500*g* for 10 min at 4°C. The supernatant was discarded. The resuspension process was repeated five times using nuclear resuspension buffer without protease inhibitor cocktail. The precipitate resuspended in 100-μL resuspension buffer was the nuclear fraction. All steps were performed at 4°C or on ice. The antibodies used in the experiments include anti-AKIN10 (1:500, Agrisera), anti-Histone H3 (1:5,000, Abcam), anti-PEPC (1:5,000, Rockland), and anti-rabbit (1:5,000, SeraCare).

### Expression and purification of recombinant SnRK1.1 and SLAH3-CT in *Escherichia coli*

Recombinant GST-SnRK1.1, GST-SLAH3-CT, GST-SLAH3-CT (S601A), and GST-SLAH3-CT (S580A) were expressed in *E. coli* (Rosetta). Protein induction and purification was performed as previously described (Cui et al., 2018). The glutathione agarose beads (Sangon Biotech) was used according to the manufacturer’s manual.

### In vitro kinase assays

For autoradiography, the experiment was performed as previously described (Lee et al., 2013).

Pro-Q diamond phosphoprotein gel stain provides a more efficient and safe method than autoradiography, and the phosphorylated proteins on the protein gel can be directly revealed by fluorescence scanner detection. The stain is compatible with a visible light-scanning instruments, imaging equipment with appropriate filters, blue-LED transilluminators, or 300-nm UV transilluminators and also can be used for mass spectrometry analysis. For this assay, the protein combinations were incubated in 20-µL kinase reaction solution containing 20 mM Tris–HCl (pH 8.0), 5 mM MgCl_2_.6H_2_O, 1 mM CaCl_2_, 1 mM DTT, and 0.5 µM ATP for 25 min at 30°C. The reaction solutions added with 5 × SDS loading buffer were boiled at 95°C for 5 min. Then they were loaded into the sodium dodecyl sulfate polyacrylamide gel electrophoresis (SDS-PAGE) gel for separating proteins. The gel was first fixed by fixation solution (50% methanol, 10% acetic acid) for 30 min. The gel was then put in fixation solution and fixed overnight. After washing twice with ddH_2_O for 15 min each time, the gel was stained with Pro-Q solution containing 1/3 volume of Pro-Q diamond phosphoprotein gel stain (Invitrogen) and 2/3 volume of ddH_2_O for 2 h in the dark. All of the following experiments were performed in dark. The gel was washed four times by destaining solution (20% acetonitrile, 50 mM sodium acetate, pH 4.0) for 30 min and washed twice by ddH_2_O for 20 min each time. Then the gel was observed with the Molecular Imager PharosFX (Bio-rad). Coomassie Brilliant Blue (CBB) staining of proteins was used for a loading control.

### Measurement of the net ${\text{NO}}_{3}^{-}$ fluxes using the NMT system

The net fluxes of ${\text{NO}}_{3}^{-}$ were measured non-invasively using the NMT with Non-invasive Micro-test system (NMT150S, YoungerUSA LLC, Amherst, MA 01002, USA) and imFluxes V2.0 (YoungerUSA LLC, Amherst, MA 01002, USA) Software. The principle of this technology and instruments has been described previously (Sun et al., 2009). Measurements were performed at room temperature (25°C). Pre-pulled and silanized 8–10 μm aperture glass ${\text{NO}}_{3}^{-}$-microsensor (XY-CGQ-01) were first filled with a backfilling solution (10 mM KNO_3_) to a length of ∼1.0 cm from the tip. LIX Holder (XY-LIX-01) was used to add NO_3_-LIX (NMT selective liquid ion-exchange cocktails, XY-SJ-NO3-25) to the front of the microelectrode. An Ag/AgCl wire microsensor holder **(**YG003-Y11**)** was inserted in the back of the microsensor to make electrical contact with the electrolyte solution used as the reference microsensor. Prior to the flux measurements, the ion selective electrode was calibrated using the calibration solution 1 (0.2 mM KNO_3_, 0.1 mM KCl, 0.1 mM CaCl_2_, 0.3 mM MES, pH 6.0) and the calibration solution 2 (2 mM KNO_3_, 0.1 mM KCl, 0.1 mM CaCl_2_, 0.3 mM MES, pH 6.0) successively. To test whether Cl^−^ interferes with the measurements of ${\text{NO}}_{3}^{-}$ in the NMT system, the ${\text{NO}}_{3}^{-}$ concentrations and net fluxes in measuring solution 1 (1 mM KNO_3_, 0.1 mM KCl, 0.1 mM CaCl_2_, 0.3 mM MES, pH 6.0) and measuring solution 2 (1 mM KNO_3_, 1.1 mM KCl, 0.1 mM CaCl_2_, 0.3 mM MES, pH 6.0) were detected without seedlings. To analyze the net ${\text{NO}}_{3}^{-}$ fluxes, the background was recorded by the vibrating electrode in measuring solution 1 without seedlings. After culturing on 1/2 solid medium for 8 d, the seedlings were treated with treatment solution (1 mM KNO_3_, 10 mM NH_4_Cl, pH 4.5) or control solution (1 mM KNO_3_, 1 mM NH_4_Cl, pH 5.7) for 2 h. Then the seedlings were transferred and fixed into small plastic dishes (6 cm diameter), and the measuring solution 1 was added into plastic dishes for measurement. The microelectrode vibrated between 5 and 35 μm from the root surface along an axis perpendicular to the root in the solution. The net fluxes of ${\text{NO}}_{3}^{-}$ were measured individually at the mature zone next to the root tip. Each plant was measured for over 6 min. The final flux values for each treatment represent the average of 3 plants. The microelectrode, LIX Holder, NO_3_-LIX, and Ag/AgCl wire microsensor holder used in the experiment were purchased from Xuyue (Beijing) Science and Technology Co., Ltd., Beijing, China.

### Mass spectrometry analysis

Protein samples separated by SDS–PAGE gels and stained with the CBB were cut to small pieces and washed twice by ddH_2_O for 10 min each time; 200-µL 100 mM NH_4_HCO_3_/ACN (1:1) was added to decolorize the gel at 37°C for 2 min. Then the samples were dehydrated by 200-µL ACN and evaporated to dryness in rotary vacuum evaporator for 5 min. The gel particles were incubated in 50-µL 10 mM TCEP (NH_4_HCO_3_ attenuating) at 56°C for 1 h, and then incubated in 50-µL 40 mM CAA (NH_4_HCO_3_ attenuating) in the dark for 45 min. Dried micelles were washed with 200-µL 25 mM NH_4_HCO_3_, 50% ACN and 100% ACN for completely dehydration. The gel particles were incubated with 50 µL ACN for 15 min, and evaporated to dryness in rotary vacuum evaporator for 5–10 min after removing ACN. Then the gel particles were incubated with 50-µL 0.01 mg/mL trypsin solution (50 mM NH_4_HCO_3_ attenuating) at 37°C overnight. The peptides were extracted with 50-µL 50% ACN/0.1% TFA twice for 10 min each time by ultrasonic instrument, evaporated to dryness in rotary vacuum evaporator and stored at −80°C. The samples were added 15-µL 0.1% FA, and centrifuged at 18000*g* for 30 min twice. The samples were analyzed by an Orbitrap Fusion Lumos mass spectrometer (Thermo Fisher Scientific) connected to an EASY-nLC 1200 system. The software Proteome Discoverer Daemon 2.2 (Thermo Fisher Scientific) was used for data analysis.

### Plant fresh weight and root length assays

Plant fresh weight for quantitative analysis was measured by Ten-thousandth electronic analytical balance. The sum of fresh weights of 5 seedlings per plate for each line was analyzed. The final values for each treatment represent the average of 3 plates. As another indicator of plant growth, primary root length was measured by the National Institutes of Health Image software ImageJ. The vertical distance between hypocotyl junction and primary root tip was defined as root length. The average of root lengths of five seedlings per plate for each line was analyzed. The final values for each treatment represent the average of three plates.

### Reproducibility and statistics

All experiments were repeated at least three times. Statistical analyses were indicated in each figure legend, and performed using the program GraphPad Prism.v8. Statistical significance is indicated by the *P*-value (*P <* *0.05; **P <* *0.01). Statistical analyses were performed by one-way analysis of variance (ANOVA with Tukey’s multiple comparisons test, two-way ANOVA analysis with Tukey’s multiple comparisons test or two-way ANOVA analysis with Dunnett’s multiple comparisons test.

### Protein structure analysis website

The topology of SLAH3 protein was predicted by http://wlab.ethz.ch/protter/start/.

### Accession numbers

Sequence data from this article can be found in the Arabidopsis Information Resource database (TAIR, http://www.arabidopsis.org/) under the following accession numbers: *SnRK1.1*, *AT3G01090*; *SnRK1.2*, *AT3G29160*; *SLAH3*, *AT5G24030*.

## Supplemental data

The following materials are available in the online version of this article.

**Supplemental Figure S1.** The sensitized emission method of FRET indicates that SnRK1.1/mSnRK1.1 interacts with SLAH3 in planta.

**Supplemental Figure S2.** Expression analyses of *SnRK1.1*/*mSnRK1.1* in *SnRK1.1*/*mSnRK1.1* overexpression lines in Col-0.

**Supplemental Figure S3.** Glucose contributes to SnRK1.1-mediated high-ammonium responses.

**Supplemental Figure S4.** Palatinose does not contribute to SnRK1.1-mediated high-ammonium responses.

**Supplemental Figure S5.** SnRK1.1 regulates ammonium toxicity depending on SLAH3.

**Supplemental Figure S6.** Expression analyses of *SLAH3* gene in *slah3-4* mutant complemented with different *SLAH3* variants and *SLAH3*.

**Supplemental Figure S7.** The phosphorylation status of S601 does not affect the interaction of SLAH3 with SnRK1.1.

**Supplemental Figure S8.** *SLAH3* can complement the sensitivity phenotype of *slah3-4* to high-ammonium/low-nitrate and low-pH condition.

**Supplemental Figure S9.** The presence of Cl^−^ does not interfere with the measurements of ${\text{NO}}_{3}^{-}$in the NMT analyses.

**Supplemental Figure S10.** SLAH3 S601 does not influence the net ${\text{NO}}_{3}^{-}$ fluxes under non-high-ammonium/low-pH condition.

**Supplemental Figure S11.** Original western blot data of Figure 10.

**Supplemental Table S1.** Primer sequences used in this study.

## Supplementary Material

kiab057_Supplementary_Data

## Notes

K.H., D.S., and S.L. conceived the project and designed the experiments; K.H., and D.S. wrote the manuscript; D.S. performed most of the experiments, X.F., K.H., C.X., X.H., Z.M., J.S., J.W., and S.W. performed the others; J.L., X.F., C.X., and S.L. helped prepare the manuscript.

The author responsible for distribution of materials integral to the findings presented in this article in accordance with the policy described in the Instructions for Authors (https://academic.oup.com/plphys/pages/general-instructions) is: Kai He (hekai@lzu.edu.cn).
