# Supplementary material for: Kinase SnRK1.1 regulates nitrate channel SLAH3 engaged in nitrate-dependent alleviation of ammonium toxicity
Source: Plant Physiol. 2021 Feb 9;186(1):731–49. doi: 10.1093/plphys/kiab057 (PMC8154061; doi:10.1093/plphys/kiab057)
Supplement: kiab057_Supplementary_Data [file kiab057_supplementary_data.docx]

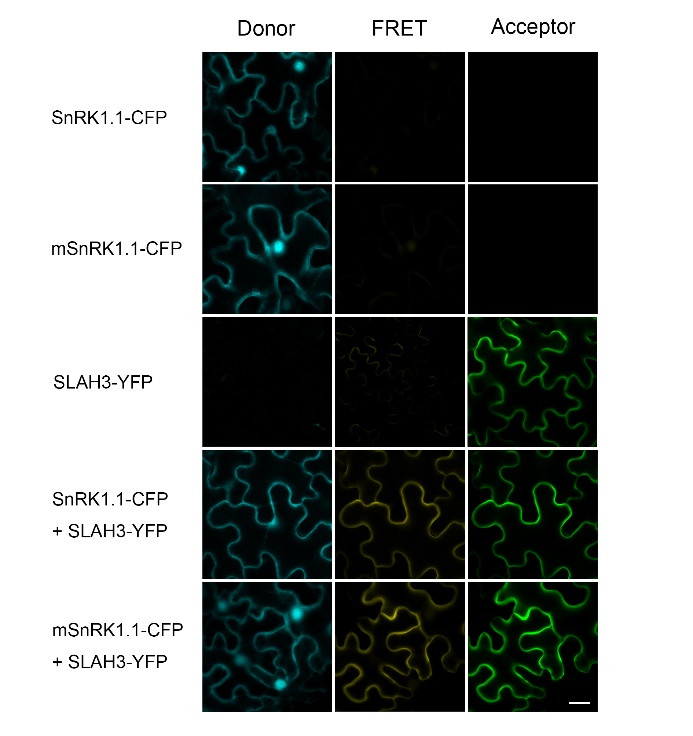


**Supplemental Figure S1.** The sensitized emission method of FRET indicates that SnRK1.1/mSnRK1.1 interacts with SLAH3 *in planta*.

The sensitized emission method of FRET was used to detect the interaction of SLAH3 with SnRK1.1/mSnRK1.1. The leaf cells of *Nicotiana benthamiana* expressing SnRK1.1/mSnRK1.1-CFP alone, SLAH3-YFP alone, and coexpressing SLAH3-YFP and SnRK1.1/mSnRK1.1-CFP and were observed. White scale = 20 µm.


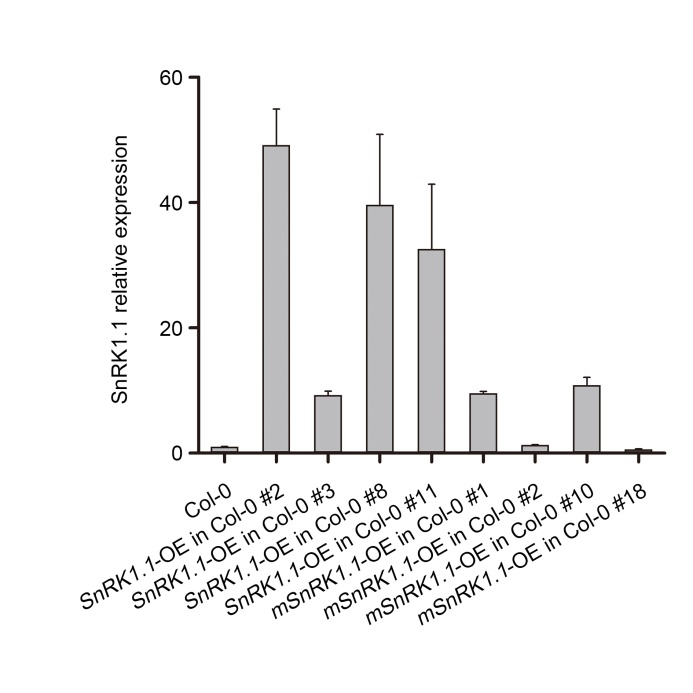


**Supplemental Figure S2.** Expression analyses of *SnRK1.1*/*mSnRK1.1* in *SnRK1.1*/*mSnRK1.1* overexpression lines in Col-0.

RT-qPCR was performed by using the total RNA extracted from 8-day-old seedlings of Col-0 and *SnRK1.1*/*mSnRK1.1* overexpression lines. Data are shown as means ± SE, *n* = 3. *SnRK1.1*-OE in Col-0 #2，#3 , and *mSnRK1.1*-OE in Col-0 #1, #10 were used for further analyses in this study.


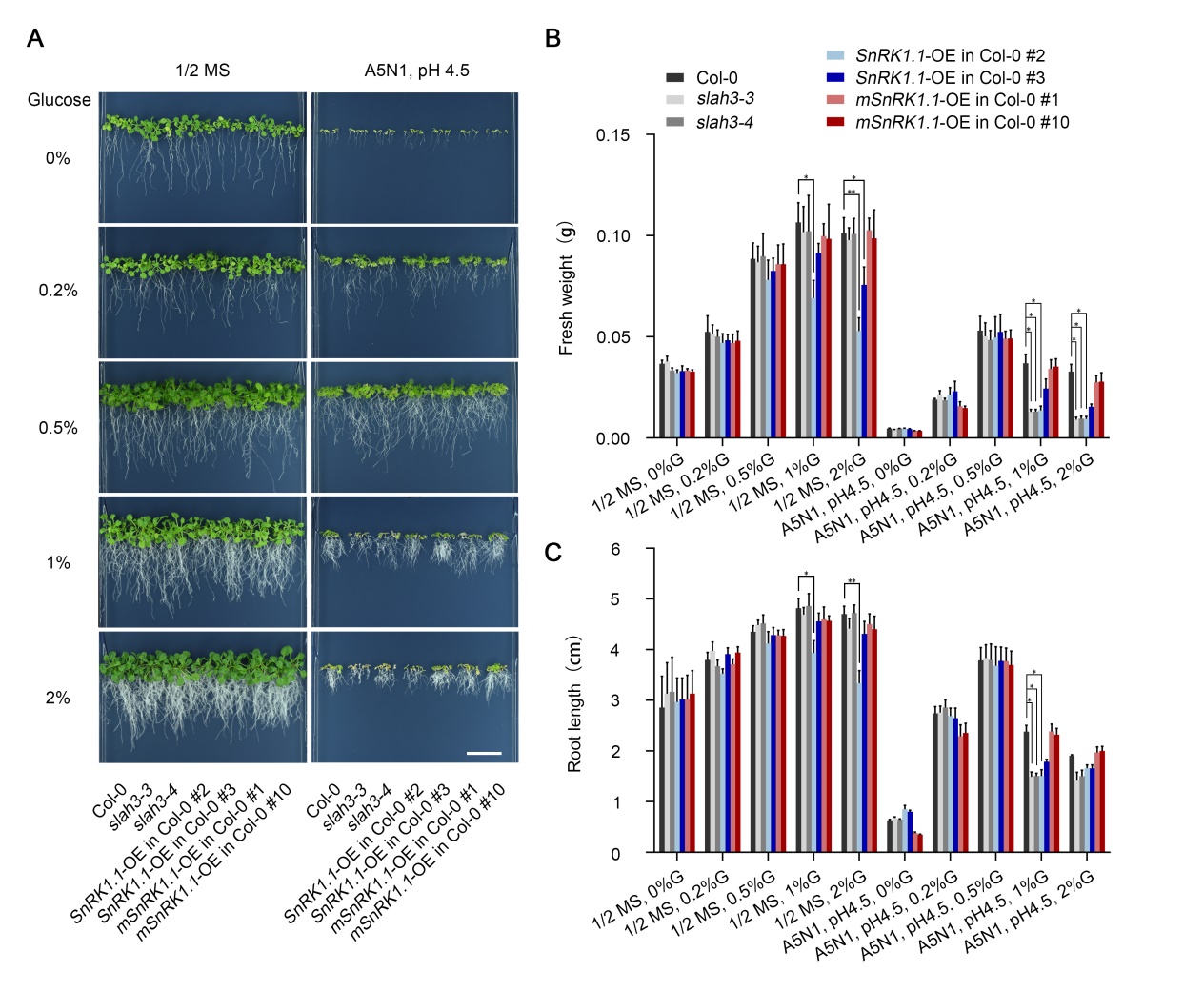


**Supplemental Figure S3.** Glucose contributes to SnRK1.1-mediated high-ammonium responses.

A, 20-day-old plants of Col-0, *slah3*, *SnRK1.1*-OE in Col-0 and *mSnRK1.1*-OE in Col-0 are presented under different concentrations of glucose in 1/2 MS and A5N1, pH 4.5 medium. White scale = 2 cm. B, The statistical analyses of fresh weight of the plants present in (A). Fresh weights of 5 seedlings per plate for each line were analyzed. Bars indicate SE, *n* = 3. C, The statistical analyses of the root length of the plants present in (A). Root lengths of 5 seedlings per plate for each line were analyzed. Bars indicate SE, *n* = 3. Statistical analyses were performed by two-way ANOVA analysis with Dunnett’s multiple comparisons test. *, *P* < 0.05; **, *P* < 0.01. G, Glucose.


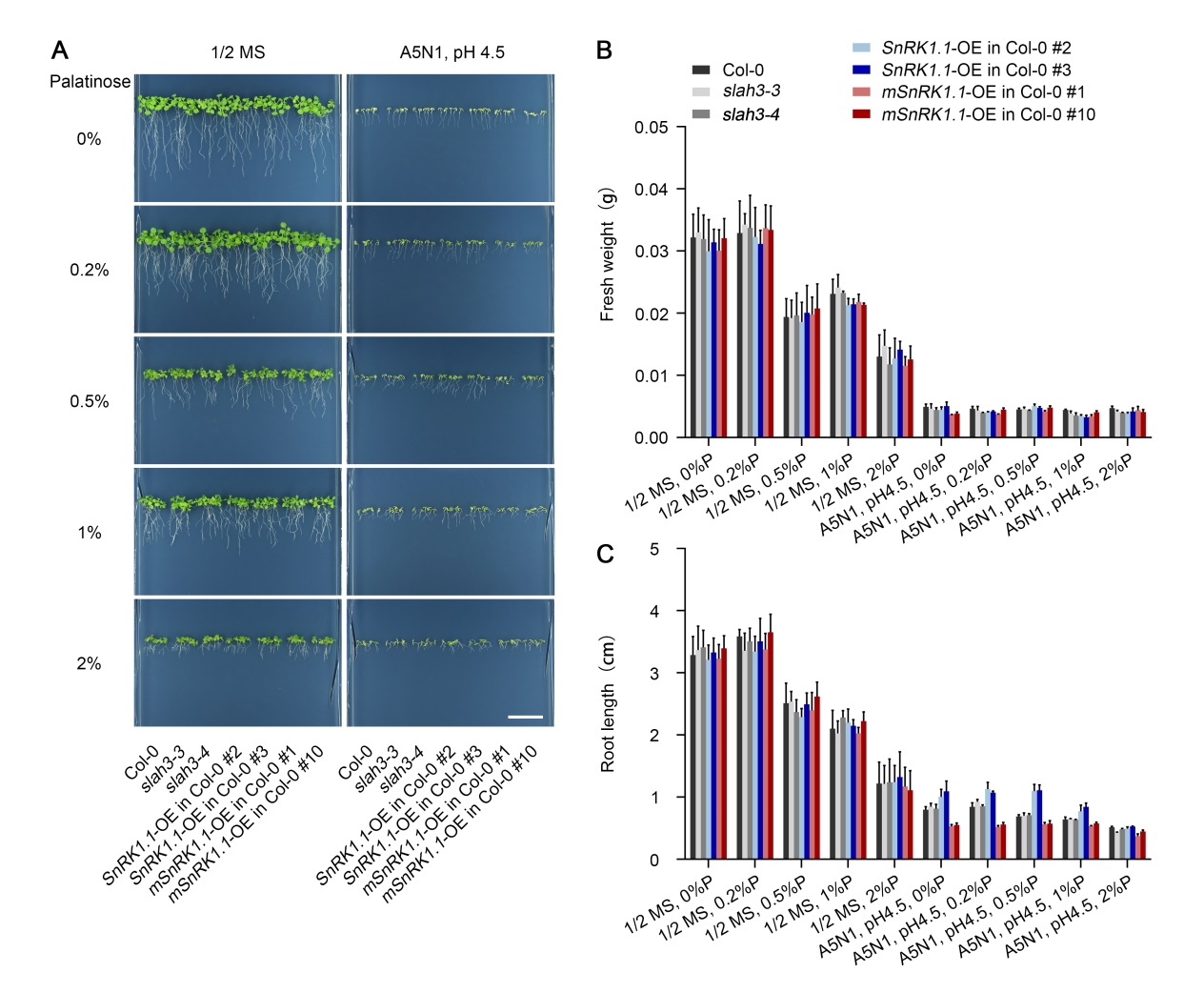


**Supplemental Figure S4.** Palatinose does not contribute to SnRK1.1-mediated high-ammonium responses.

A, 20-day-old plants of Col-0, *slah3*, *SnRK1.1*-OE in Col-0 and *mSnRK1.1*-OE in Col-0 are presented under different concentrations of non-metabolic palatinose in 1/2 MS and A5N1, pH 4.5 medium. White scale = 2 cm. B, The statistical analyses of fresh weight of the plants present in (A). Fresh weights of 5 seedlings per plate for each line were analyzed. Bars indicate SE, *n* = 3. C, The statistical analyses of the root length of the plants present in (A). Root lengths of 5 seedlings per plate for each line were analyzed. Bars indicate SE, *n* = 3. Statistical analyses were performed by two-way ANOVA analysis with Dunnett’s multiple comparisons test. *, *P* < 0.05; **, *P* < 0.01. P, Palatinose (a non-metabolizable analogue of sucrose).


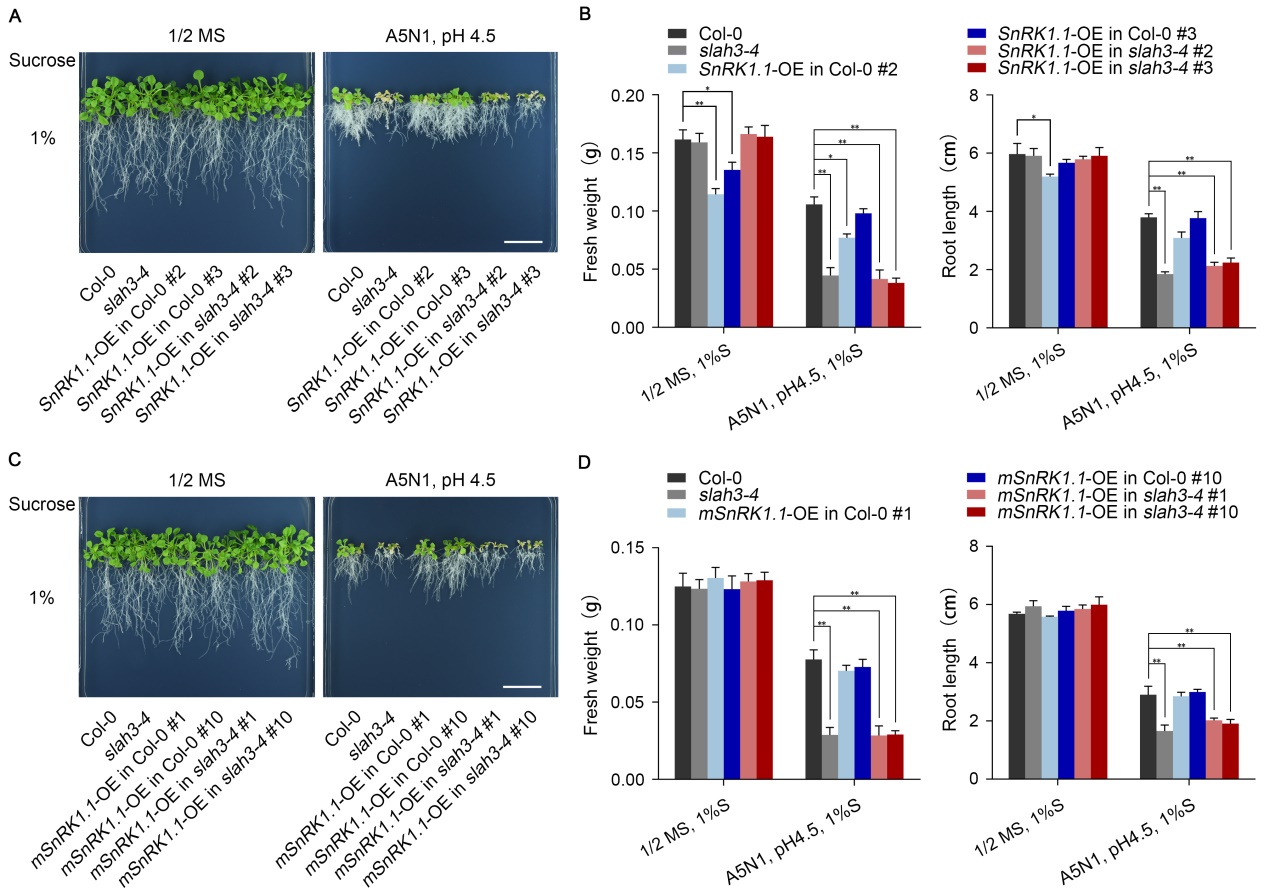


**Supplemental Figure S5.** SnRK1.1 regulates ammonium toxicity depending on SLAH3.

A and C, Phenotypes of Col-0 and *slah3-4*, *SnRK1.1* and *mSnRK1.1* overexpression plants grown for 19 days under 1/2 MS and A5N1, pH 4.5 culture conditions with 1% sucrose. Overexpression of *SnRK1.1* or *mSnRK1.1* in *slah3-4* shows similar phenotypes to *slah3-4*. White scale = 2 cm. B and D, The statistical analyses of fresh weight and root length of the plants present in **(**A**)** and (C). Fresh weights and root lengths of 5 seedlings per plate for each line were analyzed. Bars indicate SE, *n* = 3. Statistical analyses were performed by two-way ANOVA analysis with Dunnett’s multiple comparisons test. *, *P* < 0.05; **, *P* < 0.01. S, Sucrose.


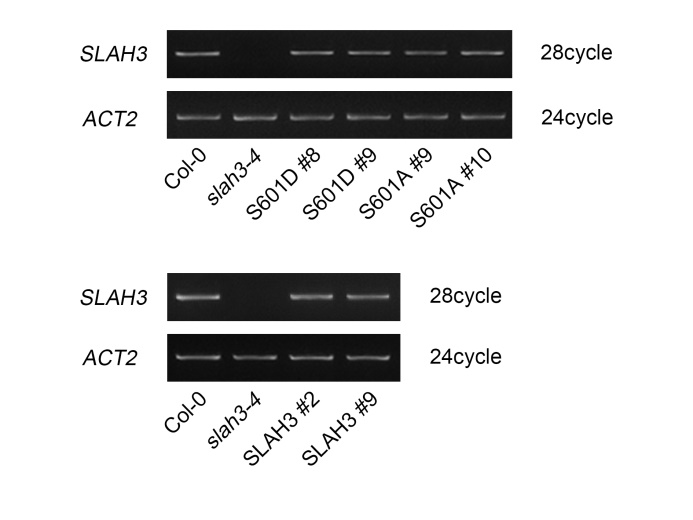


**Supplemental Figure S6.** Expression analyses of *SLAH3* gene in *slah3-4* mutant complemented with different *SLAH3* variants and *SLAH3*.

RT-PCR was performed by using the total RNA extracted from 8-day-old seedlings of Col-0, *slah3-4* and complementation lines. *SLAH3* native promoter was used to drive the expression of *SLAH3, SLAH3 S601D* (S601D) or *SLAH3 S601A* (S601A) in *slah3-4* background.


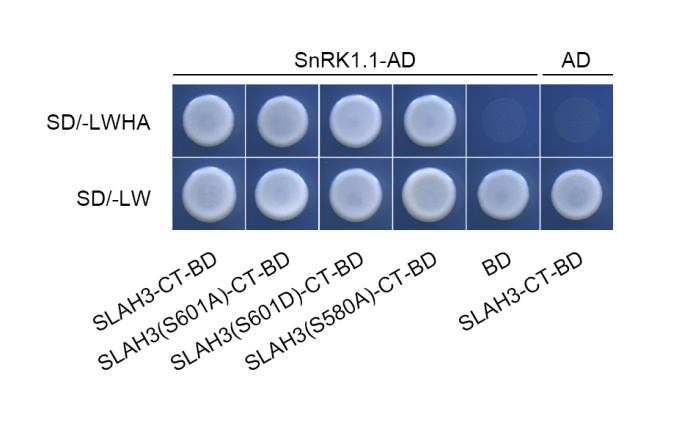


**Supplemental Figure S7.** The phosphorylation status of S601 does not affect the interaction of SLAH3 with SnRK1.1.

Co-transformed with various combinations of the plasmids were grown on synthetic dropout medium lacking Leu and Trp (-L-W) and synthetic dropout medium lacking Leu, Trp, His and adenine (-L-W-H-A). SnRK1.1 interacts with the C-terminal of SLAH3 containing *S601A* or *S601D* mutation.

**
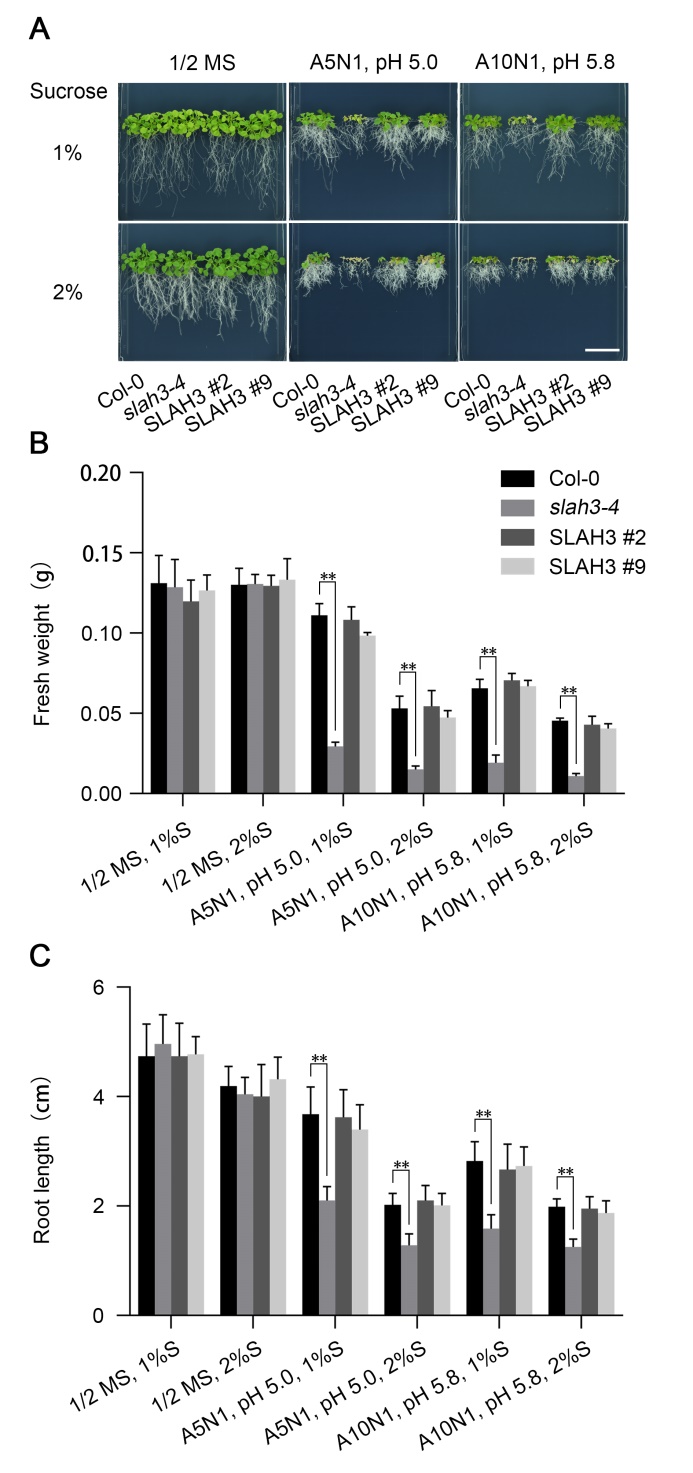
**

**Supplemental Figure S8.** *SLAH3* can complement the sensitivity phenotype of *slah3-4* to high-ammonium/low-nitrate and low-pH condition.

A, Two independent of *SLAH3* complementation lines show WT-like phenotype under high-ammonium/low-nitrate and low-pH condition. White scale = 2 cm. B and C, The statistical analyses of fresh weight **(**B**)** and root length **(**C**)** on the plants presented in (A). Data are shown as means ± SE, *n* = 3. Statistical analyses were performed by two-way ANOVA analysis with Dunnett’s multiple comparisons test. *, *P* < 0.05; **, *P* < 0.01. S, Sucrose.

**
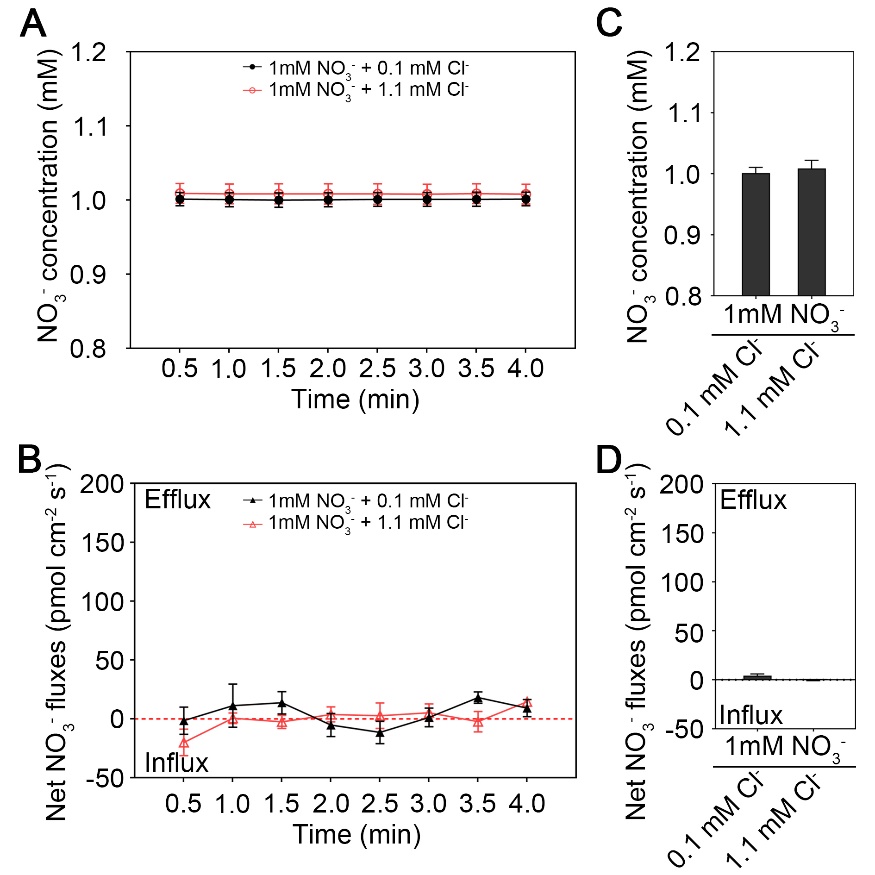
**

**Supplemental Figure S9.** The presence of Cl^-^ does not interfere with the measurements of NO_3_^-^ in the NMT analyses.

A, The NO_3_^-^ concentrations in measuring solution 1 (1 mM KNO_3_, 0.1 mM KCl, 0.1 mM CaCl_2_, 0.3 mM MES, pH 6.0) and measuring solution 2 (1 mM KNO_3_, 1.1 mM KCl, 0.1 mM CaCl_2_, 0.3 mM MES, pH 6.0) were measured by NMT method. Each point represents the mean ± SE, n = 3. B, The net NO_3_^-^ fluxes in measuring solution 1 and measuring solution 2 were measured by NMT method. Each point represents the mean ± SE, n =3. C, Mean values of NO_3_^-^ concentrations from (A) are presented. There are no significant differences. D, Mean values of NO_3_^-^ fluxes from (B) are presented. Data are shown as means ± SE, *n* = 3. Statistical analyses were performed by one-way ANOVA with Tukey’s multiple comparisons test. *P* < 0.05. There are no significant differences.

**
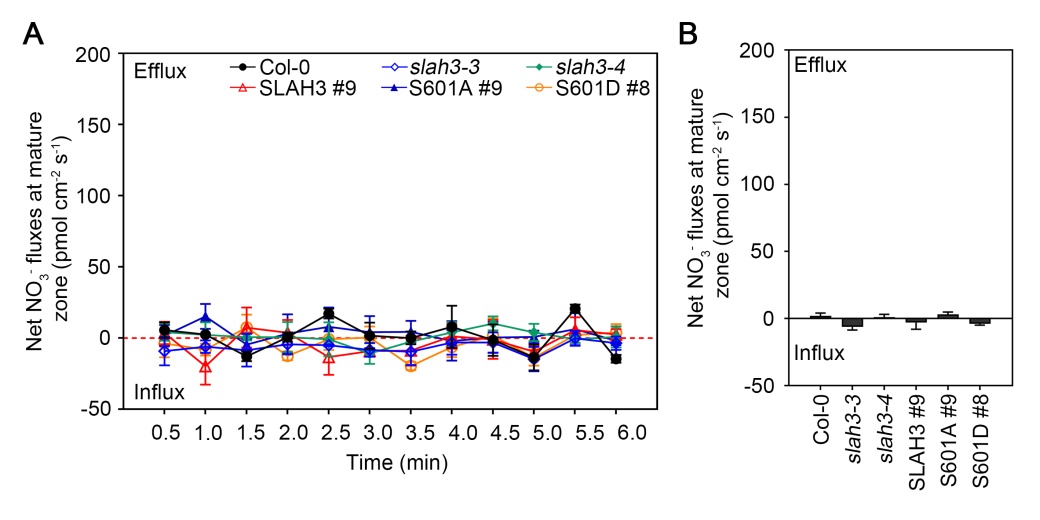
**

**Supplemental Figure S10.** SLAH3 S601 does not influence the net NO_3_^-^ fluxes under non-high-ammonium/low-pH condition.

A, The net NO_3_^-^ fluxes in Col-0, *slah3-3*, *slah3-4*, and complementary lines seedlings that were grown on 1/2 MS medium for eight days and transferred to control condition (1 mM NO_3_^-^, 1 mM NH_4_^+^, pH 5.7) for 2 h were measured by NMT method. Each point represents the mean ± SE of three individual plants. B, Mean values of NO_3_^-^ fluxes from (A) are presented. Data are shown as means ± SE, *n* = 3. Statistical analyses were performed by one-way ANOVA with Tukey’s multiple comparisons test. *P* < 0.05. There are no significant differences among all plants.

**
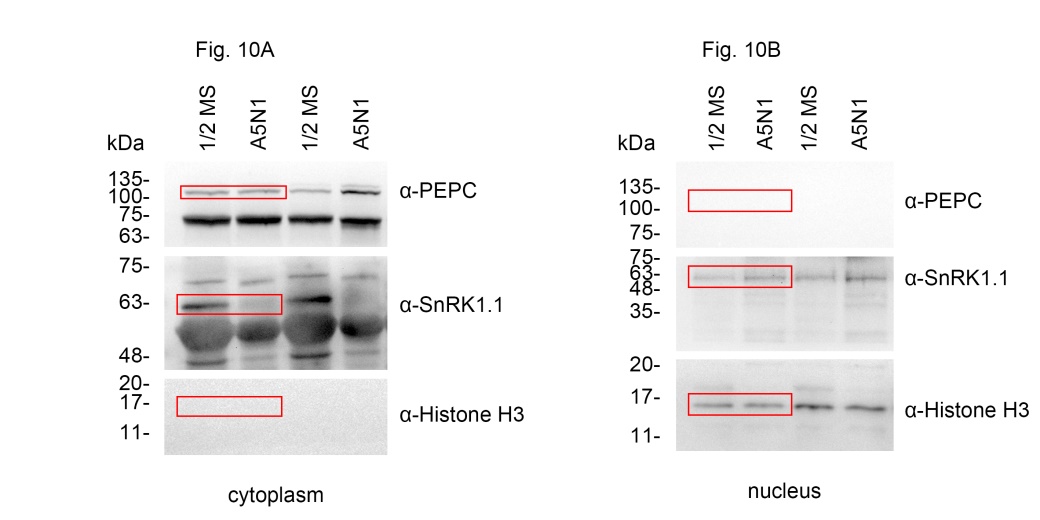
**

**Supplemental Figure S11.** Original western blot data of Figure 10.

**Supplemental Table S1.** Primer sequences used in this study.

| Primer Name | Sequence(5'→3') |
| --- | --- |
| SnRK1.1 qRT-F | CCGCTCCAGAGGTAATTTCG |
| SnRK1.1 qRT-R | CACACCACAGCTCCAGACATCT |
| attB1-SnRK1.1 pro-F | GGGGACAAGTTTGTACAAAAAAGCAGGCTTCTATCTTCTCTTTATATGTGA |
| attB2-SnRK1.1 pro-R | GGGGACCACTTTGTACAAGAAAGCTGGGTCTCTCTACAAAAAAAGAGAGAGA |
| attB1-SnRK1.1L-F | GGGGACAAGTTTGTACAAAAAAGCAGGCTTCATGTTCAAACGAGTAGATGAGT |
| attB1-SnRK1.1-F | GGGGACAAGTTTGTACAAAAAAGCAGGCTTCATGGATGGATCAGGCACAGGCA |
| attB2-SnRK1.1-R(SC) | GGGGACCACTTTGTACAAGAAAGCTGGGTCTCAGAGGACTCGGAGCTGAGCA |
| attB2-SnRK1.1-R(NSC) | GGGGACCACTTTGTACAAGAAAGCTGGGTCGAGGACTCGGAGCTGAGCA |
| SnRK1.1 K48M(+) | CATAAGGTTGCTATCATGATCCTCAATCGTCGC |
| SnRK1.1 K48M(-) | GCGACGATTGAGGATCATGATAGCAACCTTATG |
| attB1-SnRK1.1-871F | GGGGACAAGTTTGTACAAAAAAGCAGGCTTCAAAAAAGATTGACGAGGAGATT |
| attB1-SnRK1.1-1018F | GGGGACAAGTTTGTACAAAAAAGCAGGCTTCGGTTATCTCGGGGCTGAGTTTC |
| attB1-SnRK1.1-1192F | GGGGACAAGTTTGTACAAAAAAGCAGGCTTCGGGCTCATCCCCGTGAAATAAT |
| attB2-SnRK1.1-1011R(SC) | GGGGACCACTTTGTACAAGAAAGCTGGGTCTCAGGCACGGAAACGATTGTCCAGT |
| attB2-SnRK1.1-879R(SC) | GGGGACCACTTTGTACAAGAAAGCTGGGTCTCAAATCTTTTTTGCCTGTTGCACA |
| attB2-SnRK1.1-1401R(SC) | GGGGACCACTTTGTACAAGAAAGCTGGGTCTCAGGGCGACTTAACAGCTGCTTCG |
| attB1-SLAH3 pro-F | GGGGACAAGTTTGTACAAAAAAGCAGGCTTCTTGCGATATCAGTCTTATGT |
| attB2-SLAH3 pro-R | GGGGACCACTTTGTACAAGAAAGCTGGGTCCTGACGATTTTGAAATTTTG |
| attB1-SLAH3-F | GGGGACAAGTTTGTACAAAAAAGCAGGCTTCATGGAGGAG AAACCAAACTA |
| attB2-SLAH3-R(SC) | GGGGACCACTTTGTACAAGAAAGCTGGGTCTTATGATGAATCACTCTCTT |
| attB1-SLAH3 C559-F | GGGGACAAGTTTGTACAAAAAAGCAGGCTTCACCACCATTATCCACGCCTT |
| attB2-SLAH3 N253-R | GGGGACCACTTTGTACAAGAAAGCTGGGTCTCACGGCCACTTTTTATCATT |
| SLAH3 S580A(+) | CCTCGCCATAGCCATCGCCAACCGTCCGAGACCCA |
| SLAH3 S580A(-) | TGGGTCTCGGACGGTTGGCGATGGCTATGGCGAGG |
| SLAH3 S601A(+) | CCAACTGAGAAACGTAGCCTCAGAGAACATCGAGAACT |
| SLAH3 S601A(-) | AGTTCTCGATGTTCTCTGAGGCTACGTTTCTCAGTTGG |
| SLAH3 S601D(+) | CCAACTGAGAAACGTAGACTCAGAGAACATCGAGAACT |
| SLAH3 S601D(-) | AGTTCTCGATGTTCTCTGAGTCTACGTTTCTCAGTTGG |
| SLAH3 S601E(+) | CCAACTGAGAAACGTAGAATCAGAGAACATCGAGAACT |
| SLAH3 S601E(-) | AGTTCTCGATGTTCTCTGATTCTACGTTTCTCAGTTGG |
| SLAH3 RT-F | GCCACAACAGAAGAAATGGTTG |
| SLAH3 RT-R | CCGTTGCAAGCTTCTACATC |
| *slah3-3* LP | ATTGTCTCTCAAGTTTCCCCG |
| *slah3-3* RP | GGTCAGATTTATTTAATTATCGGCC |
| *slah3-4* LP | CTACCGACCAACGAGACTTTG |
| *slah3-4* RP | TGCTATTTATACAGTGATAACCCCTC |
| LBb1.3 | ATTTTGCCGATTTCGGAAC |
| ACT2 qRT-F | CCCGATGGGCAAGTCATC |
| ACT2 qRT-R | GAACAAGACTTCTGGGCATCTGA |
| ACT2 RT-F | GCGGATCCATGGCTGAGGCTGATGATATTCAACC |
| ACT2 RT-R | CGTCTAGACCATGGAACATTTTCTGTGAACGATTCC |
| 35S-F | CCTTCGCAAGACCCTTCCTC |
| GUS-R | ATCCAGACTGAATGCCCACA |
